# Enantioselective synthesis of [1,1′-binaphthalene]-8,8′-diyl bis(diphenylphosphane) and its derivatives†

**DOI:** 10.1039/d3ra07956b

**Published:** 2024-01-17

**Authors:** Yao Tang, Qingkai Yuan, Sai Zhang, Jia-Yin Wang, Kazimierz Surowiec, Guigen Li

**Affiliations:** a Department of Chemistry and Biochemistry, Texas Tech University Lubbock Texas 79409-1061 USA guigen.li@ttu.edu; b School of Pharmacy, Continuous Flow Engineering Laboratory of National Petroleum and Chemical Industry, Changzhou University Changzhou Jiangsu 213164 China

## Abstract

Two 8,8′ disubstituted binaphthyl ligands have been designed and synthesized in 3.1% and 11.4% overall yield, respectively. X-ray structure analysis demonstrated that a unique chiral microenvironment was created. With the assistance of a new aggregation-induced polarization (AIP) technology, chiral aggregates were determined as the fraction of polar solvent increased in the nonpolar/polar solvent system, which indicated their potential in modern asymmetric synthesis and catalysis.

## Abstract 2

A new chiral phosphine ligand and its diol derivative have been designed and synthesized asymmetrically.

## Graphical abstract

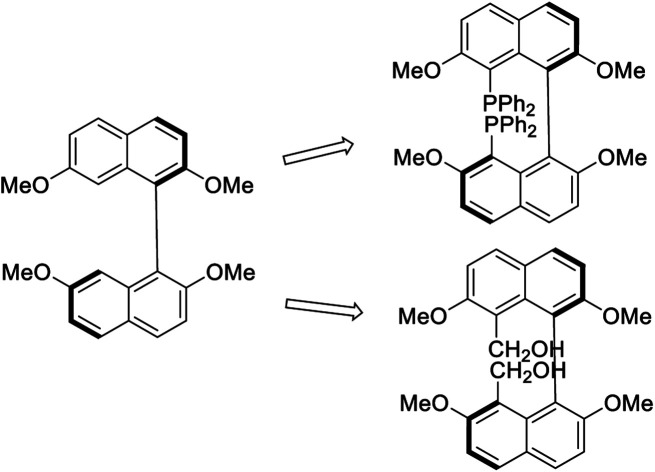

## Introduction

In recent decades, the metal catalytic asymmetric reaction has been well developed for various purposes in pharmaceuticals and agrochemicals.^1,2^ Bidentate ligands, such as diphosphate and diol ligands, are commonly applied due to their strong coordination affinity to the metal. Among these ligands, *C*_2_ symmetric biphenyl and binaphthyl ligands with a restricted axis can provide an excellent chiral environment in metal-chiral scaffolds.^3^ As a representative, chiral 2,2′-bis(diphenylphosphino)-1,1′-binaphthyl (BINAP) and chiral 1,1′-bi-2-naphthol (BINOL) and their derivatives have been widely applied as chiral ligands in asymmetric Suzuki–Miyaura coupling reaction, asymmetric hydrogenation, metal catalytic Mannich reaction, and C(sp_2_)–H bond functionalization and gave promising results.^3–6^ Regioisomeric ligands with electron-donating groups substituted at different positions on the biaryl backbone have been identified with identical asymmetric control.^7^ This control correlates to the changes in bite angle and the chiral environment created in the metal complex.^8^ However, the modification of BINAP and BINOL by introducing the electron-donating groups to other locations on the binaphthyl has not been extensively reported. Much of the progress was impeded by low yield and enantioselectivity after chiral resolution and the multistep synthesis required to get the desired structures.^3,9^ Zhang and coworkers developed bidentate phosphine-phosphoramidite ligands that introduced two diphenylphosphine groups at the 3,3′-positions of the chiral BINOL backbone. The resulting ligands demonstrated highly effective enantiomeric control on the hydrogenation of α-aryl enamide.^10^ Shibata and coworkers investigated the catalytic performance of a series of BINOL-PHOS with the phosphorous group at 3,3′, 4,4′, and 6,6′ positions of the BINOL architecture and determined that identical regioisomeric ligands can give opposite enantioselective products in the Cu-catalyst asymmetric conjugated addition reaction.^7,11^

The 2,2′ and 8,8′ positions of binaphthalene always play a crucial role in assembling the atropisomers generally found in natural drugs and chiral ligands.^12^ The functional groups attached at the 2,2′ position significantly increase the rotational barriers, preventing atropisomerization at elevated temperatures.^13,14^ The peri-positioned functional groups at the 8,8′ position on the binaphthyl backbone can affect the topology and provide a unique microenvironment for asymmetric transformation.^15–17^ Herein, we report the enantioselective synthesis of two new *C*_2_ symmetric 8,8′-disubstituted binaphthyl ligands *via* chiral resolution of racemic 7,7′-dimethoxy-[1,1′-binaphthalene]-2,2′-diol. The final products can be obtained in 3.1% and 11.4% yields in total *via* two more step transformations.

## Results and discussion

**Scheme 1:**
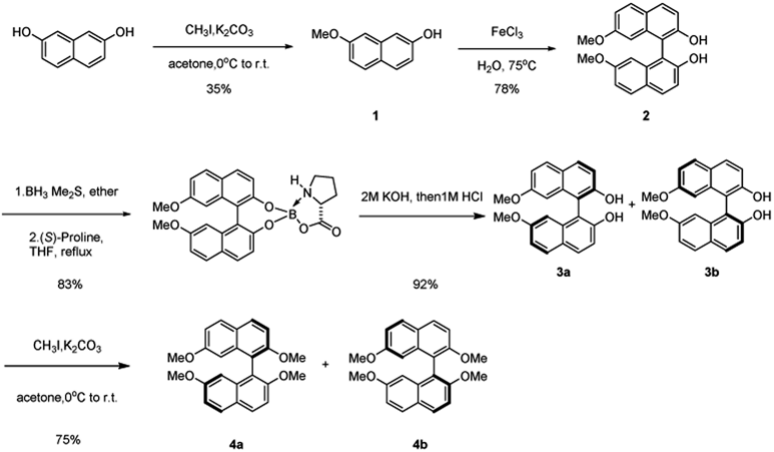
Synthesis and chiral resolution of (±) 4.

The asymmetric synthesis of the two new types of 8,8′-bidentate ligands begins with the chiral resolution of (±) 4 (Scheme 1), which is initiated by the partial methylation of commercially available naphthalene-2,7-diol in the presence of methyl iodide and potassium carbonate as the reaction temperature gradually increases from 0 °C to room temperature, giving 7-methoxynaphthalen-2-ol 1 in 35% yield. Many metal salts were reported as effective catalysts for the self-oxidative coupling of naphthol derivatives. Herein, FeCl_3_·H_2_O was selected as the coupling reagent for constructing racemic 7,7′-dimethoxy-[1,1′-binaphthalene]-2,2′-diol 2, resulting in 78% yield under 75 °C overnight.^18^ The chiral resolution step follows Shan's protocol, which treated the binaphthalene compound with borane dimethyl sulfide complex and (*S*)-proline.^19^ The two diastereomers can be successfully separated by column chromatography. The deprotection step was achieved by treatment with 2 M KOH followed by 1 M HCl to give enantiopure (*R*)-7,7′-dimethoxy-[1,1′-binaphthalene]-2,2′-diol 3a and (*S*)-7,7′-dimethoxy-[1,1′-binaphthalene]-2,2′-diol 3b. The protection of free diol from either 3a or 3b was performed by using an excess amount of potassium carbonate and methyl iodine in the presence of acetone as solvent. After the reaction was completed, the precursors 4a and 4b of the 8,8′-bidentate ligands can be recrystallized by methanol with 75% yield.

**Scheme 2:**
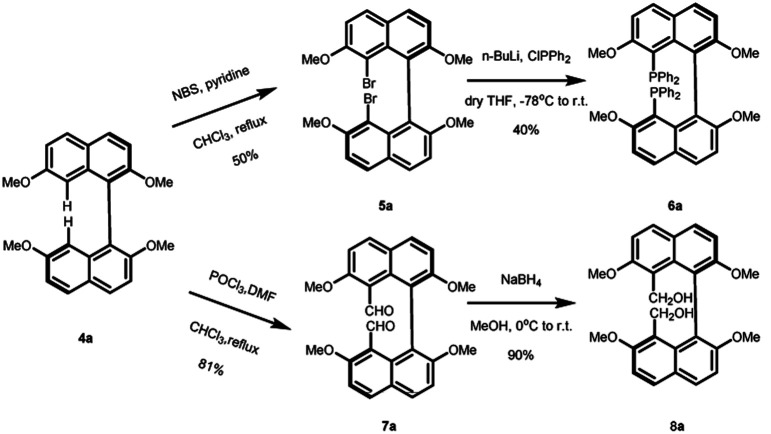
Synthesis of ligands 6a and 8a.

With the enantiopure (*R*)-2,2′,7,7′-tetramethoxy-1,1′-binaphthalene in hand, we next conducted the synthesis of new bidentate ligands bearing diphosphine and dihydroxy as the electron-donating groups by two more steps, with recrystallization as the only purification technology (Scheme 2). Precursor 4a was chosen as the substrate for the following synthetic work. The diphosphine ligand 6a was assembled by bromination using *N*-bromosuccinimide (NBS) under reflux. The double-brominated product 5a was treated with *n*-BuLi in anhydrous THF at −78 °C, followed by the dropwise addition of chlorodiphenylphosphine, and the reaction temperature gradually increased to room temperature. The reaction was quenched by 1 M HCl to give diphosphine ligand in 50% yield. The synthesis of dihydroxy ligand 8a was begun by a well-known Vilsmeier–Haack formylation to give the (*R*)-8,8′-dicarbaldehyde in 81% yield. Next, the final product was obtained in 90% yield by NaBH_4_ reduction.

**Fig. 1:**
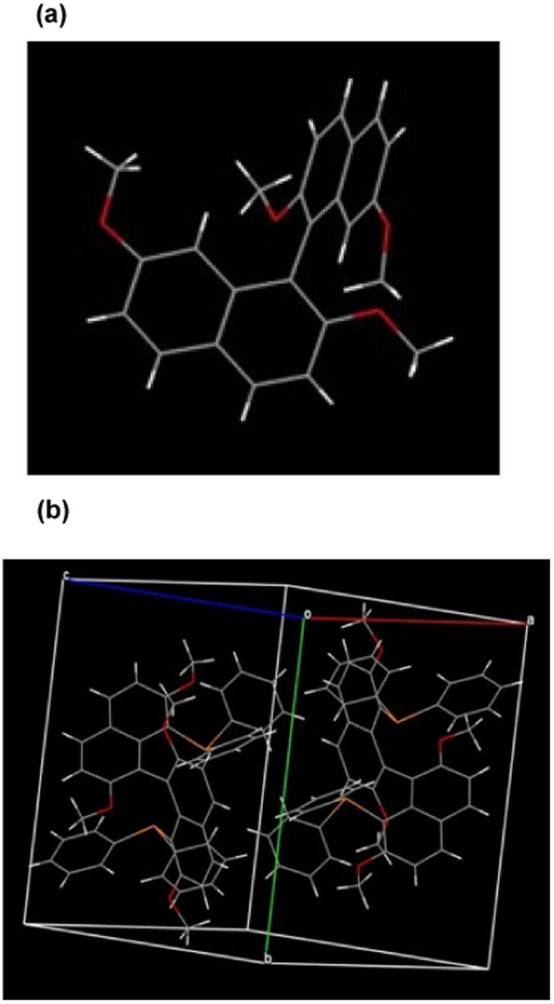
X-ray structural analysis of product 3a and racemic 6. (a) Absolute configuration determination of molecule 3a. (b) Crystal unit cell containing racemic molecule 6.

To our delight, single crystals of molecule 3a and racemic 6 were obtained by slow evaporation of the toluene solution containing each sample, respectively. As the X-ray reveals, the absolute configuration of 3a is defined as *R*, and the torsion angle between the naphthalene planes is 106.96° (Fig. 1a). Furthermore, the two substituted diphenylphosphine groups increase the steric effect at the 8,8′-position, featuring a slightly increased torsion angle (118.87°). Compared to the traditional 2,2′-bis(diphenylphosphino)-1,1′-binaphthyl (BINAP) ligands, this newly designed ligand has a larger torsion angle attributed to the induced steric and electronic features by tetramethoxy groups (Fig. 1b). Interestingly, one of the aromatic rings from the diphenylphosphine group is almost perpendicular to its substituted naphthalene, and the other phenyl ring from the same diphenylphosphine ring is nearly parallel to the counter naphthyl. The particular spatial arrangement between the diphenylphosphine and binaphthyl groups can create an effective chiral environment and make the asymmetric catalytic reaction feasible.

**Fig. 2:**
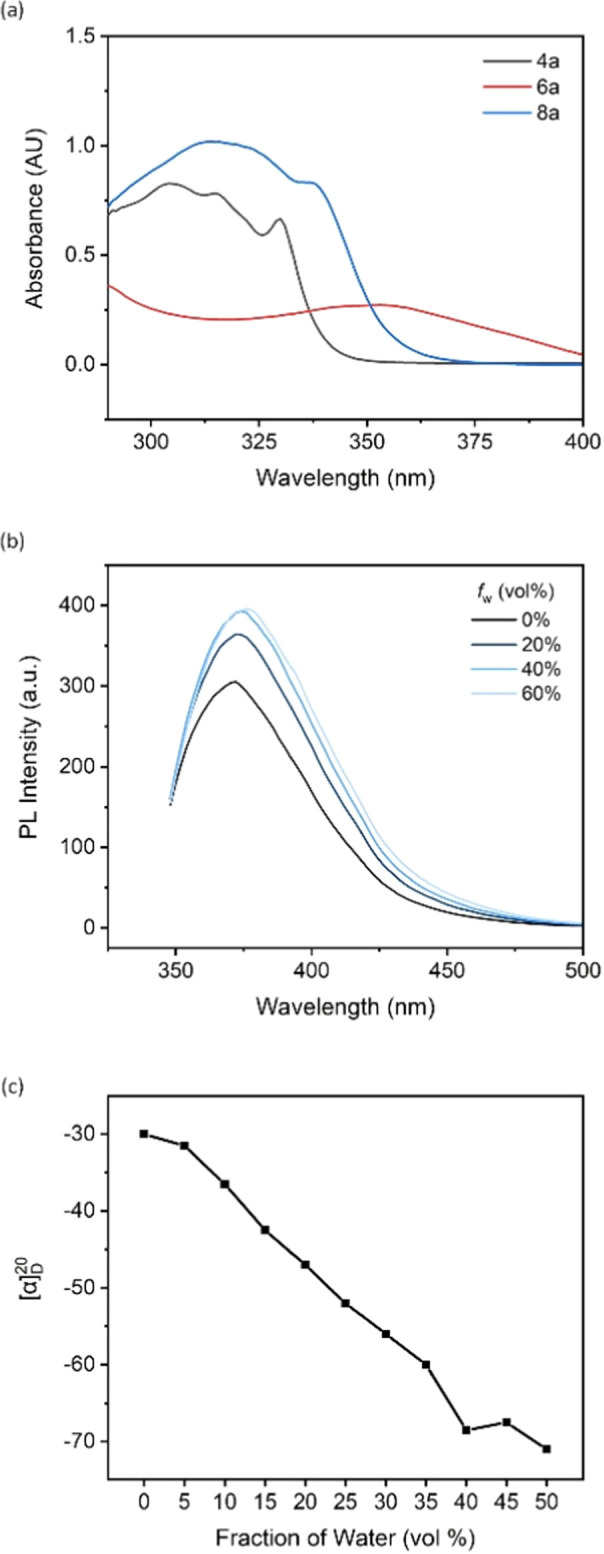
(a) UV/vis absorption of 4a, 6a and 8a in THF; *c* = 25 μg mL^−1^. (b) PL spectra of 4a in THF/water mixtures with different water fractions (*f*_w_); *c* = 0.1 mM; *λ*_ex_ = 342 nm. (c) Aggregation-induced polarization (AIP) of 8a in THF/water cosolvent; *c* = 2 mg mL^−1^.

The UV-vis absorption study of compound 4a and ligands 6a and 8a was performed by dissolving in THF. Compound 4a and ligand 6a exhibited very similar absorptive behaviors, featuring maximum absorptions at around 305 nm and 315 nm, respectively (Fig. 2a). Compared to substrate 4a, ligand 6a showed the highest redshift, moving from 305 nm to 354 nm, which can be attributed to the electron-donating effect from the two diphenylphosphine groups. In addition, the tight packing of disubstituted diphenylphosphine groups at the 8,8′ positions of the binaphthyl backbone induced a conformational π–π interaction, which is more likely to be the reason for the highly bathochromic effect.

A photoluminescence (PL) study of 4a was performed to investigate its aggregation-induced emission (AIE) behavior in THF/water mixture. By adjusting the irradiation power to 500 V and excitation wavelength to 342 nm, the emission intensity increased from 305 to 364 as the water fraction changed from 0% to 20% (Fig. 2b). This enhancement supports the AIE effect existing in the new tetramethoxy binaphthyl architecture, and polar solvent is more likely to restrict the free rotation of the aromatic rings, resulting in dissipation of the excited-state energy. The fluorescence intensity peaked at 393 as the *f*_w_ reached 40%, and the intensity remained consistent as water was added.

Chiral aggregates have been determined as a novel tool for controlling chiral induction in the asymmetric GAP (group-assisted purification) synthesis of the 2,3-dihydrobenzofuran series. The stereoselectivity of the final product can be inverted by changing the predominant solvent in typical AIE cosolvents (nonpolar solvents/polar solvents).^20^ However, the development of this new technology is hampered by the lack of detection tools for monitoring the formation of chiral aggregates. Recently, our group reported a new analytical method, aggregation-induced polarization (AIP), based on the linear relationship between the optical rotation value of the chiral samples and increasing fraction of protonic solvent in the solvent mixture. This technique screened a series of BINOL and BINAP derivatives, and AIP phenomena were successfully observed in all cases.^21,22^ Therefore, ligand 8a was selected as the representative for the AIP investigation. All measurements were finalized *via* Rudolph polarimeter (Rudolph Research Analytica APIV/2W) with samples prepared at the same concentration as *c* = 2 mg mL^−1^ under a 598 nm sodium lamp. The specific rotation values demonstrated a descending trend as the water fraction consistently increased by 5% in the THF/H_2_O mixture. The value rose slightly from −68.5° to −67.5° as the water fraction increased from 40% to 50%, then decreased to −71° when the water fraction reached 50% (Fig. 2c). These results illustrated that the chiral aggregates formed when the protic solvent gradually became the predominant one in the cosolvent system, and the application of ligand 8a in chiral aggregation-based catalysis will be studied by this group soon.

## Conclusions

In summary, two new 8,8′-bidentate ligands have been synthesized *via* chiral resolution followed by two more transformation steps in 3.1% and 11.4% yield, respectively. The absolute configuration of the binaphthyl precursor was determined, and X-ray analysis revealed a unique chiral microenvironment of the disubstituted diphenylphosphine ligand. AIE phenomenon was also found *via* photoluminescence study, and the complementary AIP study supported the existence and formation of chiral aggregates as the fraction of polar solvent increased. All results indicated that the two 8,8′-bidentate ligands would play an important role in organic synthesis, especially in chiral aggregate-based asymmetric synthesis and catalysis. The application of these chiral ligands on multilayer and orientational chirality and THF/H_2_O-based AIS^23–27^ will be conducted in due course.

## Conflicts of interest

The authors declare no conflict of interest.

## Supplementary Material

RA-014-D3RA07956B-s001
