# Supplementary material for: Enantioselective synthesis of [1,1′-binaphthalene]-8,8′-diyl bis(diphenylphosphane) and its derivatives
Source: RSC Adv. 2024 Jan 17;14(4):2792–5. doi: 10.1039/d3ra07956b (PMC10792274; doi:10.1039/d3ra07956b)
Supplement: RA-014-D3RA07956B-s001 [file RA-014-D3RA07956B-s001.pdf]

Electronic Supplementary Material (ESI) for RSC Advances.
This journal is © The Royal Society of Chemistry 2024

Supporting Information

Enantioselective Synthesis of [1,1'-Binaphthalene]-8,8'-diyl)bis(diphenylphosphine) and Derivatives

Yao Tang†, Qingkai Yuan†, Sai Zhang†, Jia-Yin Wang‡, Kazimierz Surowiec† and Guigen Li†,*

†Department of Chemistry and Biochemistry, Texas Tech University, Lubbock, Texas 79409-1061, USA.
‡School of Pharmacy, Continuous Flow Engineering Laboratory of National Petroleum and Chemical Industry, Changzhou University, Changzhou, Jiangsu 213164, China.

Context

1. General Information……………………………………………………………………S2
2. Synthetic Procedures of racemic compounds……………………………………..……...S3-S5
3. Synthetic Procedures of chiral compounds………………………………………..……...S6-S9
4. NMR Spectra ……………….…………………………………………………...…….. S10-S20
5. X-ray data ……………………………………………………………………………… S21-S31
6. HPLC spectrum …………………..S32-S38
7. Reference ………………………………………………………………………………..S39

1. General Information

Unless otherwise stated, all reactions were magnetically stirred and conducted in oven-dried glassware in anhydrous solvents under Ar. Heated oil baths were used for reactions requiring elevated temperatures. Solvents were removed under reduced pressure at 40-65 °C using a rotavapor. All given yields are isolated yields of chromatographically and NMR spectroscopically materials.

All commercially available chemicals were used as received without further purification. Solvents as follows: MeOH, EtOH, toluene, DMF, EtOAc, DCM, hexane, THF, acetone and 1,4-dioxane were used without further purification.

The ^{1}H and ^{13}C NMR spectra were recorded in $CDCl_3$ on 400 MHz and 100 MHz instruments with TMS as internal standard. For referencing of the ^{1}H NMR spectra, the residual solvent signal ($\delta = 7.26$ for $CDCl_3$) was used. In the case of the ^{13}C NMR spectra, the signal of solvents ($\delta = 77.06 \pm 0.03$ for $CDCl_3$ and $\delta = 39.52$ for DMSO-d_6) were reported in ppm with respect to TMS. Data are represented as follows: chemical shift, multiplicity (s = singlet, d = doublet, t = triplet, m = multiplet), coupling constant (*J*, Hz), and integration.

X-ray data were collected on a Rigaku XtaLAB Synergy-*i* Kappa diffractometer equipped with a PhotonJet-*i* X-ray source operated at 50 W (50kV, 1 mA) to generate Cu Kα radiation (λ = 1.54178 Å) and a HyPix-6000HE HPC detector. Crystals were transferred from the vial and placed on a glass slide in polyisobutylene. A Zeiss Stemi 305 microscope was used to identify a suitable specimen for X-ray diffraction from a representative sample of the material. The crystal and a small amount of the oil were collected on a MiTeGen 100 micron cryoloop and transferred to the instrument where it was placed under a cold nitrogen stream (Oxford 700 series) at 100K.The sample was optically centered with the aid of a video camera to ensure that no translations were observed as the crystal was rotated through all positions. A unit cell collection was then carried out. After it was determined that the unit cell was not present in the CCDC database a data collection strategy was calculated by *CrysAlis^{Pro}*[1]. The crystal was measured for size, morphology, and color.

2. Synthetic Procedures of racemic compounds

2.1. Synthesis of **[1,1'-binaphthalene]-2,2',7,7'-tetraol**

[1,1'-binaphthalene]-2,2',7,7'-tetraol was synthesized from naphthalene-2,7-diol following the reported procedure [1] with very minor modification.

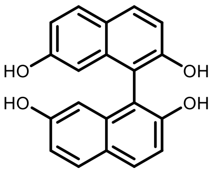

2.2. Synthesis of **2,2',7,7'-tetramethoxy-1,1'-binaphthalene** (**±4**)

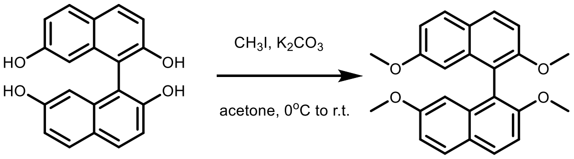

±4

[1,1'-binaphthalene]-2,2',7,7'-tetraol (318 mg, 1 mmol), K_2CO_3 (1104 mg, 8 mmol) and 20 mL acetone were added to a 50 mL round flask, which was equipped with a stirring bar, rubber septum at 0°C. The flask was degassed under vacuum and backfilled with argon 3 times and then (1.7 g, 12 mmol) CH_3I was added drop by drop. The reaction was slowly warmed to room temperature and stirred overnight. White solid **±4** can be obtained by recrystallizing in methanol (263mg, 70%).

^{1}H NMR (400 MHz, CHLOROFORM-D) δ 7.85 (t, J = 8.1 Hz, 2H), 7.73 (dd, J = 9.8, 4.4 Hz, 2H), 7.31 – 7.26 (m, 2H), 6.97 (dd, J = 8.9, 2.5 Hz, 2H), 6.42 (d, J = 2.5 Hz, 2H), 3.75 (d, J = 3.6 Hz, 6H), 3.49 (s, 6H).

2.3. Syntheses of **±4**, **±6**, and **±8**

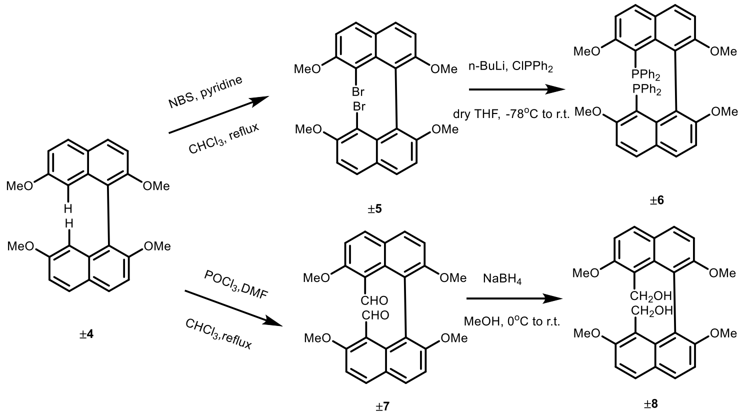

2.3.1. Synthesis of ±**6**

±**4** (383 mg, 1 mmol), NBS (720 mg, 4 mmol) and 15mL $CHCl_3$ were added in a 50 mL round flask, which was equipped with an air condenser. The flask was degassed under vacuum and backfilled with argon 3 times, and then pyridine (0.24 mL, 3 mmol) was added. The mixture was stirred at 75 ℃ under Ar overnight and quenched by 10 mL1M HCl. The reaction mixture was diluted with CH_2Cl_2 (30 mL) and washed with 20 mL brine. The organic phase was concentrated and purified by recrystallization with methanol, giving a brown solid ±**5** (0.27g, 50% yield). ±**5** can be used without further purification.

±**5** (533 mg, 1 mmol) was dissolved in 10 mL dry THF in a 50 mL Schlenk flask. The flask was degassed under vacuum and backfilled with argon 3 times. And then the mixture was stirred at -78℃ for 0.5 h and 2 mL1.6M n-BuLi was added dropwise. 20 mins later, Ph_2PCl (0.58 mL, 4 mmol) was added drop by drop. 3 h later, the reaction was slowly warmed to room temperature and stirred overnight. After completion of the reaction, the mixture was extracted with DCM. The residue was dried by rotavapor and recrystallized by using methanol to give yellow solid ±**6** (298 mg, 40%).

^{1}H NMR (400 MHz, CHLOROFORM-D) δ 7.80 (dd, *J* = 33.8, 7.9 Hz, 2H), 7.54 (d, *J* = 8.4 Hz, 2H), 7.23 (m, 10H), 6.95 (d, *J* = 8.5 Hz, 2H), 6.86 – 6.70 (m, 10H), 6.33 (d, *J* = 8.7 Hz, 2H), 2.92 (d, *J* = 12.4 Hz, 12H).

^{31}P NMR (162 MHz, CHLOROFORM-D) δ -14.61.

2.3.2. Synthesis of ±**7**

4 mL phosphorus oxychloride ($POCl_3$) and 4 mL DMF were placed in a 50 mL round bottom flask, which was equipped with a rubber septum. The flask was put into an ice bath for 15 minutes with stirring, and ±**4** (766 mg, 2 mmol) dissolved in 6 mL $CHCl_3$. Then S1 solution was added into it drop by drop. The ice bath was removed, and the flask was equipped with an air condenser and heated to 75°C for 12h, then cooled and poured into ice water. The pH of the mixture was changed to 7 by adding dilute KOH solution, and the precipitate was filtered to get crude product ±**7**.

^{1}H NMR (400 MHz, CHLOROFORM-D) δ 9.34 (t, *J* = 11.3 Hz, 2H), 7.95 – 7.85 (m, 4H), 7.22 (dd, *J* = 9.0, 3.5 Hz, 2H), 7.11 (dd, *J* = 9.1, 3.4 Hz, 2H), 3.80 (t, *J* = 5.6 Hz, 6H), 3.74 (t, *J* = 6.0 Hz, 6H).

2.3.3. Synthesis of ±**8**

±**7** (130 mg, 0.3 mmol), $NaBH_4$ (114 mg, 3 mmol) and 15 mL methanol were added into a 50 mL round bottle flask. The flask was fitted with a balloon and stirred around 8 h. After completion of the reaction, the mixture was quenched by adding water. Then the solution was extracted with EA and washed by brine and water. The organic layer was concentrated by rotavapor and recrystallized in methanol to get crude product ±**8.**

.

^{1}H NMR (400 MHz, DMSO-D6) δ 7.88 (dd, *J* = 9.1, 3.1 Hz, 4H), 7.28 – 7.21 (m, 4H), 4.22 (dd, *J* = 11.1, 6.4 Hz, 2H), 4.06 (dd, *J* = 11.1, 3.5 Hz, 2H), 3.79 (d, *J* = 3.4 Hz, 6H), 3.73 (dd, *J* = 6.3, 3.9 Hz, 2H), 3.45 (s, 6H).

3. Synthetic Procedures of chiral compounds

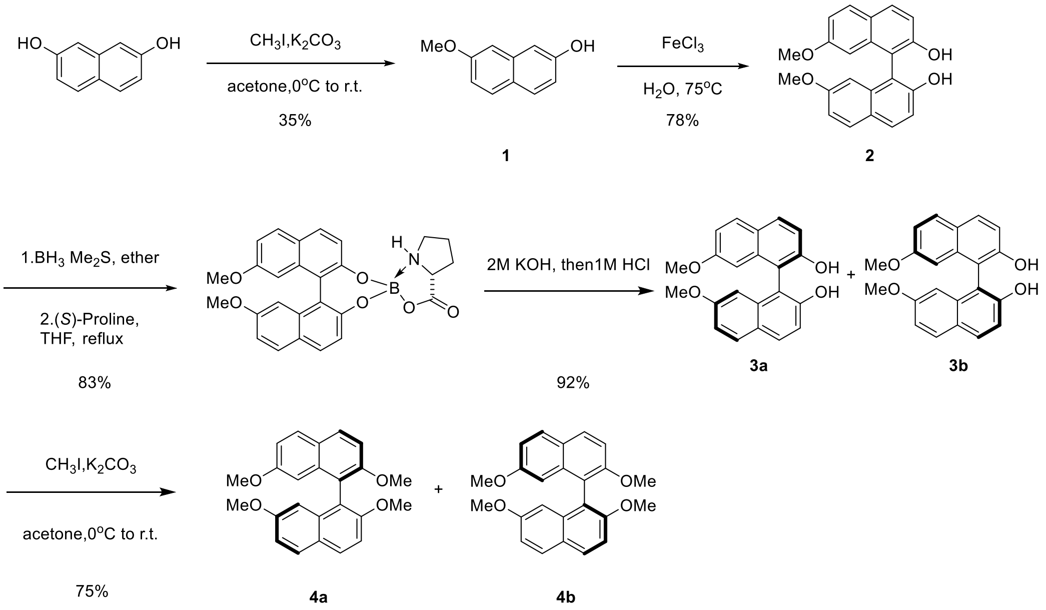

3.1. Synthesis of **7-methoxynaphthalen-2-ol** (**1**)

Naphthalene-2,7-diol (8 g, 50 mmol), K_2CO_3 (6.9 g, 50 mmol) and 100 mL acetone were added to a 250 mL round flask, which was equipped with a stirring bar, rubber septum at 0°C. The flask was degassed under vacuum and backfilled with argon 3 times and then CH_3I (3.74 mL, 60 mmol) was added drop by drop. The reaction was slowly warmed to room temperature and stirred overnight. After completion of the reaction, ethyl acetate (100 mL) and dilute ammonium hydroxide solution (20 mL) were added into the reaction mixture. Then the organic layer was washed by brine (100 mL) and dried over Na_2SO_4. The solvent was dried by rotavapor and purified by silica column (eluent Ethyl acetate: Hexane=20:1) to get white solid **1** (3.05g, 35%).

^{1}H NMR (400 MHz, CHLOROFORM-D) δ 7.64 (dd, J = 9.2, 3.9 Hz, 2H), 7.06 – 6.88 (m, 4H), 4.88 (s, 1H), 3.89 (s, 3H).

3.2. Synthesis of **7,7'-dimethoxy-[1,1'-binaphthalene]-2,2'-diol** (**2**)

7-methoxynaphthalen-2-ol (3.48 g, 20 mmol), ferric chloride hexahydrate (10.8 g, 40 mmol) and 120 mL water were added to a 250 mL round flask, which was equipped with a stirring bar and an air condenser. The reaction was heated to 75 °C and stirred overnight. After completion of the reaction, ethyl acetate (100 mL) was added into the reaction mixture. Then the organic layer was washed by brine (100 mL) and dried over Na_2SO_4. The solvent was dried by rotavapor and purified by silica column (eluent Ethyl acetate: Hexane=20:1) to get white solid **2** (2.71 g, 78%).

^{1}H NMR (400 MHz, CHLOROFORM-D) δ 7.86 (d, J = 8.8 Hz, 2H), 7.76 (t, J = 7.3 Hz, 2H), 7.21 (d, J = 8.8 Hz, 2H), 7.01 (dt, J = 10.2, 5.1 Hz, 2H), 6.47 (d, J = 2.5 Hz, 2H), 5.02 (d, J = 14.8 Hz, 2H), 3.56 (s, 6H).

3.3 Syntheses of **3a** and **3b**

3a and **3b** were synthesized from **2** following the reported procedure [2] with very minor modification.

HPLC conditions: Daicel Chrialpak IC column; hexane/2-propanol = 90/10, 1 mL/min, Retention times: 10.360 min (R), 14.913 min (S).

3.4 Syntheses of **4a** (or **4b**)

3a (or **3b**) (346 mg, 1 mmol), K_2CO_3 (552 mg, 4 mmol) and 20 mL acetone were added to a 50 mL round flask, which was equipped with a stirring bar, rubber septum at 0°C. The flask was degassed under vacuum and backfilled with argon 3 times and then CH_3I (852 mg, 6 mmol) was added drop by drop. The reaction was slowly warmed to room temperature and stirred overnight. White solid **4a** (or **4b**) can be obtained by recrystallizing in methanol (281mg, 75%). HRMS (ESI-TOF) *m/z* [$C_{24}H_{22}O_4$ +Na]$^+$ calcd for 397.1415, found 397.139sub-D1.9, *c* = 0.34 (4a), CH_2Cl_2

4a ^{1}H NMR (400 MHz, CHLOROFORM-D) δ 7.86 (d, *J* = 8.9 Hz, 2H), 7.76 – 7.71 (m, 2H), 7.31 – 7.26 (m, 2H), 6.97 (dd, *J* = 8.9, 2.5 Hz, 2H), 6.42 (d, *J* = 2.5 Hz, 2H), 3.75 (s, 6H), 3.49 (s, 6H).

^{13}C NMR (101 MHz, CHLOROFORM-D) δ 158.14, 155.59, 135.25, 129.57, 129.19, 124.93, 118.68, 116.07, 111.59, 103.87, 56.85, 55.07.

HPLC conditions: Daicel Chrialpak IC column; hexane/2-propanol = 95/5, 1 mL/min, Retention times: 6.813 min (R), 7.497 min (S).

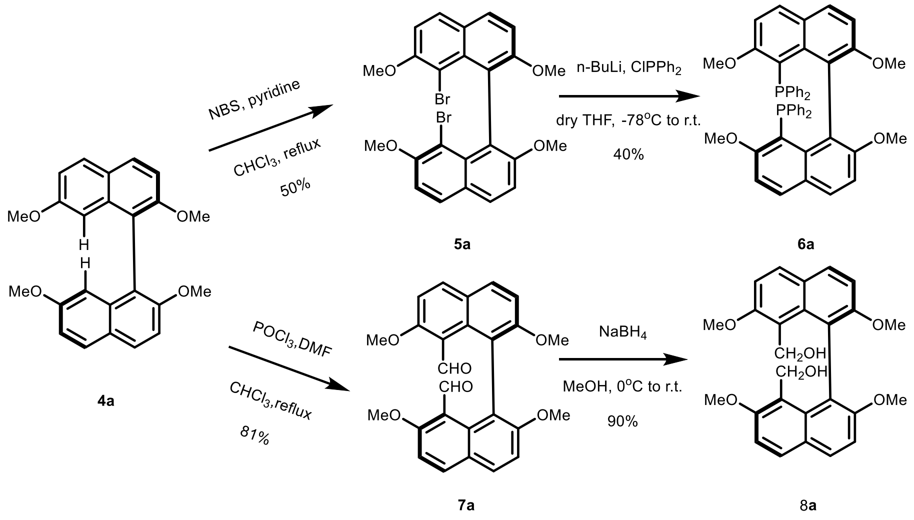

3.5 Synthesis of **6a**

4a (383 mg, 1 mmol), NBS (720 mg, 4 mmol) and 15mL $CHCl_3$ were added in a 50 mL Schlenk flask. The flask was degassed under vacuum and backfilled with argon 3 times and then pyridine (0.24 mL, 3 mmol) was added. The mixture was stirred at 75 ℃ under argon overnight and quenched by 10 mL1M HCl. The reaction mixture was diluted with CH_2Cl_2 (30 mL) and washed with 20 mL brine. The organic phase was concentrated and purified by recrystallization with methanol, giving a brown solid **5a** (0.27g, 50% yield). **5a** can be used without further purification.

HPLC conditions: Daicel Chrialpak IC column; hexane/2-propanol = 85/15, 1 mL/min, Retention times: 13.770 min (S), 39.543 min (R).

5a (533 mg, 1 mmol) was dissolved in 10 mL dry THF in a Schlenk flask. The flask was degassed under vacuum and backfilled with argon 3 times. And then the mixture was stirred at -78℃ for 0.5 h and 2 mL1.6M *n*-BuLi was added drop wisely. 20 mins later, Ph_2PCl (0.58 mL, 4 mmol) was added drop by drop. 3 h later, the reaction was slowly warmed to room temperature and stirred overnight. After completion of the reaction, the mixture was extracted with DCM. The residue was dried by rotavapor and recrystallized by using methanol to give yellow solid **6a** (300 mg, 40%). HRMS (ESI-TOF) *m/z* $[C_{48}H_{40}O_4P_2 +H]^+$ calcd for 743.2480, found 743.2455. $[\alpha]_D^{RT}$= +57.9, *c* = 0.32, CH_2Cl_2

^{1}H NMR (400 MHz, CHLOROFORM-D) δ 7.80 (dd, *J* = 33.8, 7.9 Hz, 2H), 7.54 (d, *J* = 8.4 Hz, 2H), 7.23 (m, 10H), 6.95 (d, *J* = 8.5 Hz, 2H), 6.86 – 6.70 (m, 10H), 6.33 (d, *J* = 8.7 Hz, 2H), 2.92 (d, *J* = 12.4 Hz, 12H).

^{13}C NMR (101 MHz, CHLOROFORM-D) δ 162.06, 154.57, 145.01, 142.53, 136.53, 135.04, 133.35, 129.57, 127.34, 127.00, 126.70, 125.04, 124.81, 122.30, 111.82, 110.68, 54.93.

3.6 Synthesis of **7a**

4 mL phosphorus oxychloride ($POCl_3$) and 4 mL DMF were placed in a 50 mL round bottom flask, which was equipped with a rubber septum. The flask was put into an ice bath for 15 minutes with stirring, and **4a** (766 mg, 2 mmol) dissolved in 6 mL $CHCl_3$. The **4a** solution was added into it drop by drop. The ice bath was removed, and the flask was equipped with an air condenser and heated to 75°C for 12h, then cooled and poured into ice water. The pH of the mixture was changed to 7 by adding dilute KOH solution, and the precipitate was filtered to get crude product **7a** (700 mg, 81%). HRMS (ESI-TOF) *m/z* $[C_{26}H_{22}O_6 +Na]^+$ calcd for 453.1314, found 453.1292. $[\alpha]_D^{RT}$= +127.8, *c* = 0.46, CH_2Cl_2

^{1}H NMR (400 MHz, CHLOROFORM-D) δ 9.36 (s, 2H), 7.95 – 7.86 (m, 4H), 7.21 (d, *J* = 9.0 Hz, 2H), 7.11 (dd, *J* = 8.9, 5.1 Hz, 2H), 3.81 (d, *J* = 2.2 Hz, 6H), 3.74 (s, 6H).

^{13}C NMR (101 MHz, CHLOROFORM-D) δ 193.09, 157.46, 156.66, 134.07, 133.43, 131.84, 124.75, 122.08, 118.09, 111.49, 110.92, 56.59, 56.36.

3.7 Synthesis of **8a**

7a (130 mg, 0.3 mmol), $NaBH_4$ (114 mg, 3 mmol) and 15 mL methanol were added into a 50 mL round bottle flask. The flask was fitted with a balloon and stirred around 8 h. After completion of the

reaction, the mixture was quenched by adding water. Then the solution was extracted with EA and washed by brine and water. The organic layer was concentrated by rotavapor and recrystallized in methanol to get whit solid **8a** (117 mg, 90%). HRMS (ESI-TOF) *m*/*z* [$C_{26}H_{26}O_6$ +Na]$^+$ calcd for 457.1627, found 457.1607. $[\alpha]_D^{RT}$= -56.9, *c* = 0.33, CH_2Cl_2

^{1}H NMR (400 MHz, CHLOROFORM-D) δ 7.92 – 7.85 (m, 4H), 7.26 – 7.23 (m, 2H), 7.19 (d, *J* = 9.0 Hz, 2H), 4.38 (d, *J* = 12.2 Hz, 2H), 4.31 – 4.26 (m, 2H), 3.90 (s, 6H), 3.62 (s, 6H).

^{13}C NMR (101 MHz, CHLOROFORM-D) δ 157.25, 156.63, 136.34, 130.71, 129.77, 125.67, 122.35, 120.30, 112.86, 111.83, 57.82, 57.28, 56.96.

4. NMR Spectra

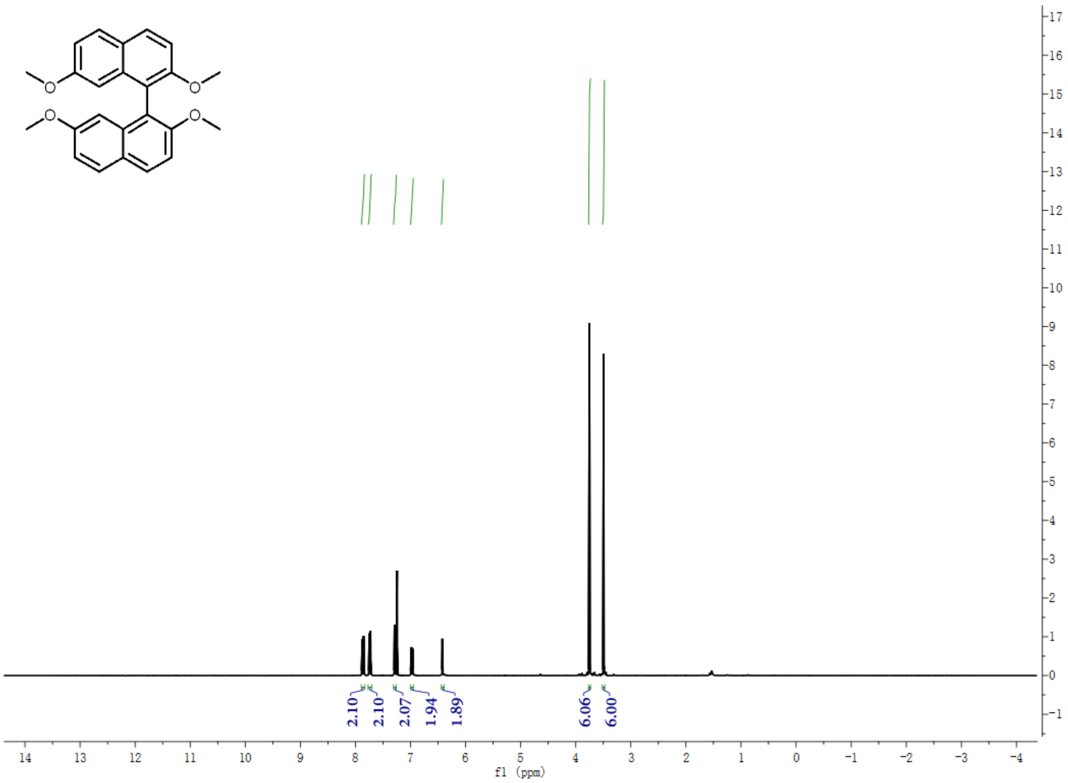

Figure S1. ^{1}H NMR spectrum of **[1,1'-binaphthalene]-2,2',7,7'-tetraol**

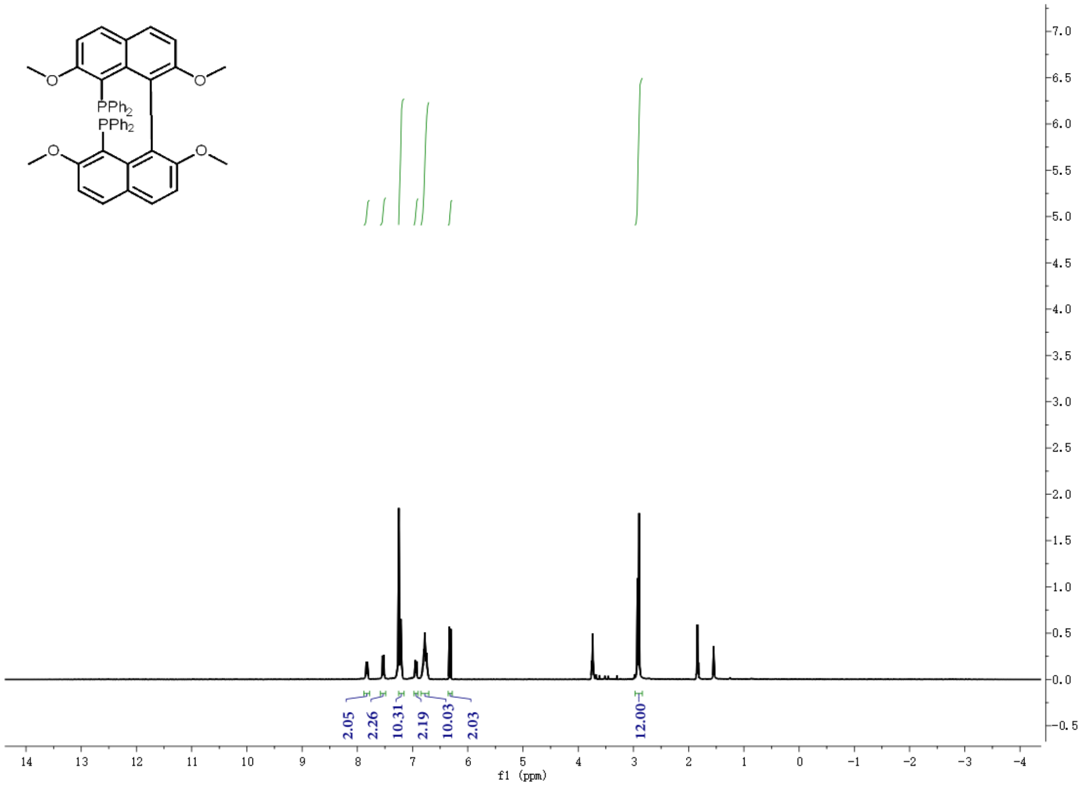

Figure S2. ^{1}H NMR spectrum of ±**6**

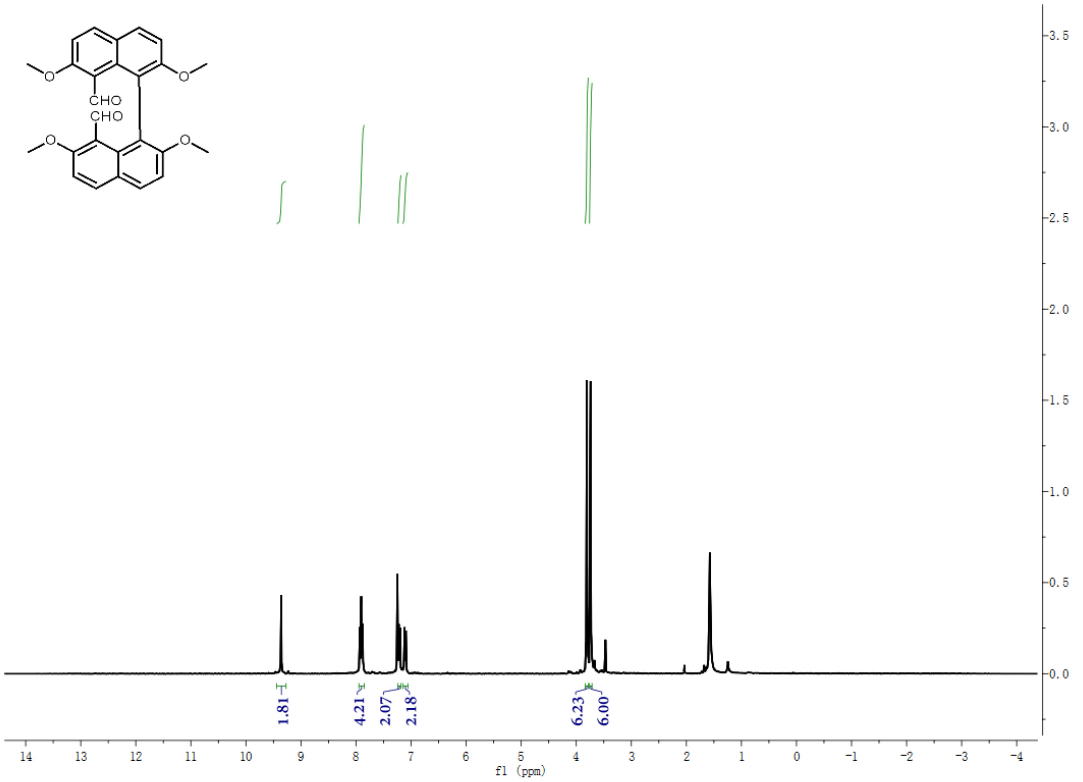

Figure S3. ^{1}H NMR spectrum of ±**7**

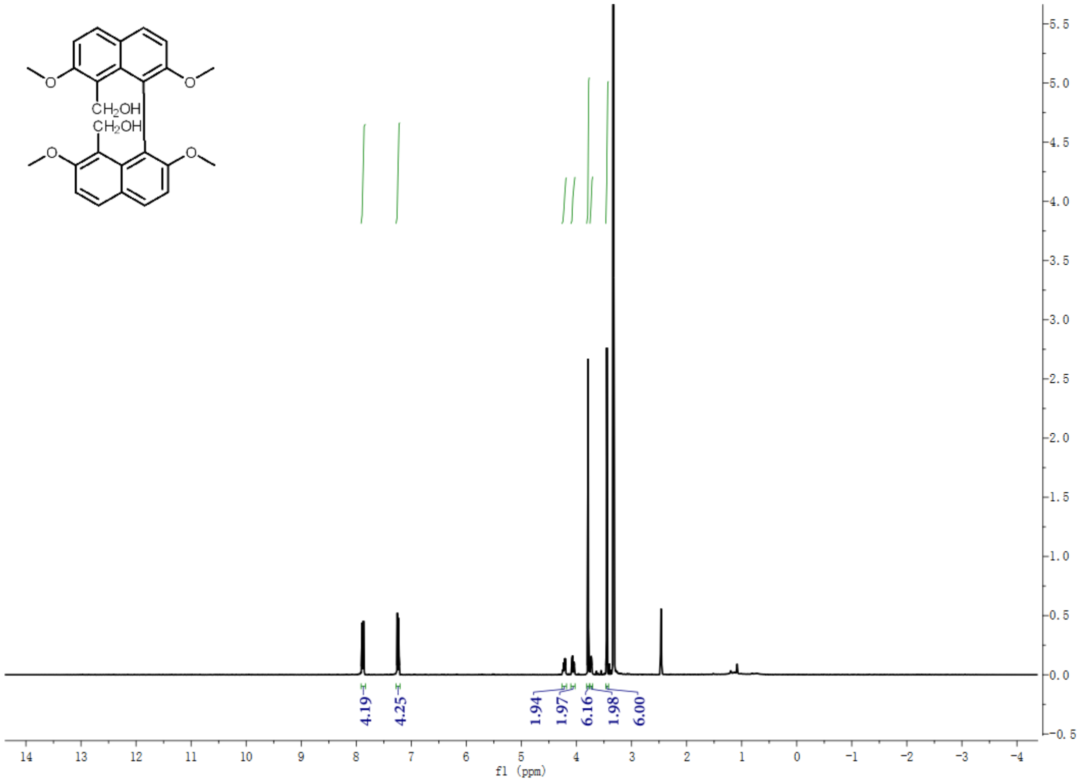

Figure S4. ¹H NMR spectrum of **±8**

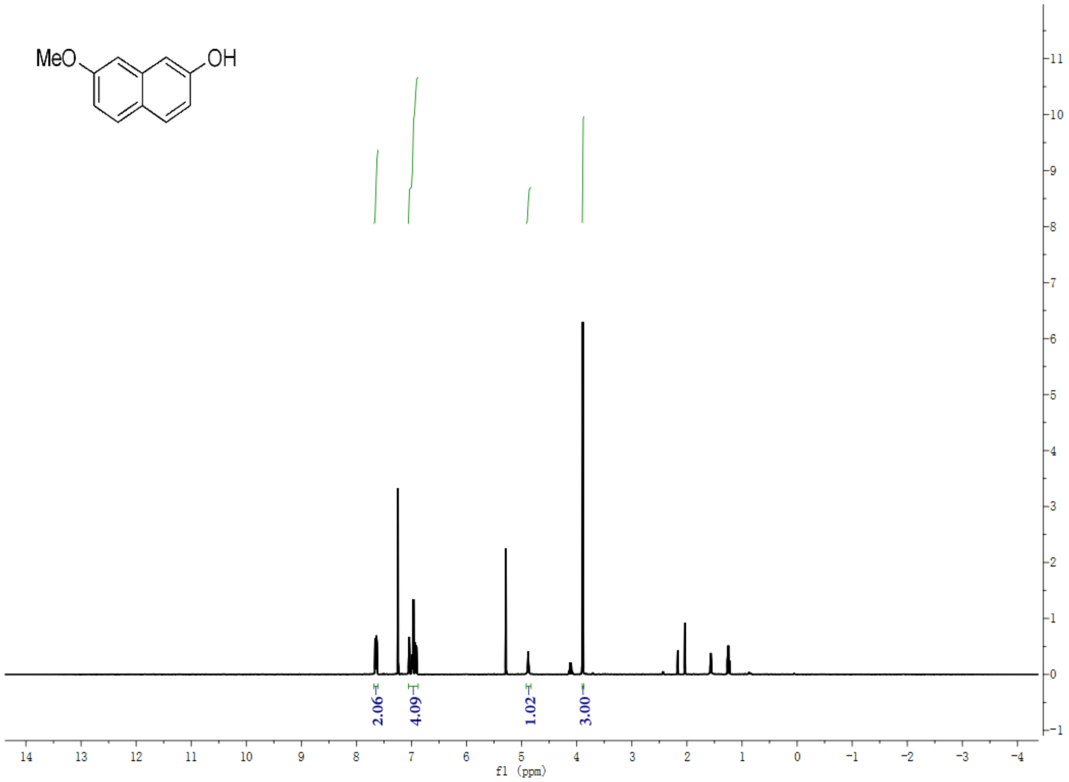

Figure S5. ^{1}H NMR spectrum of **1**

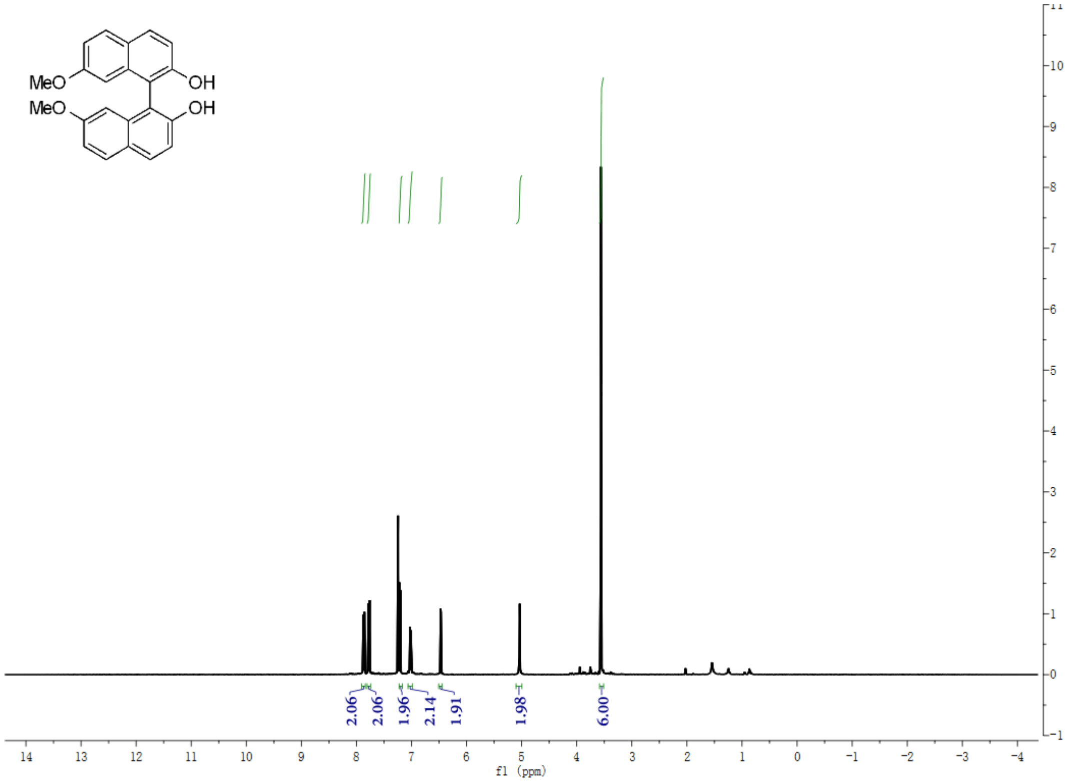

Figure S6. ^{1}H NMR spectrum of **2**

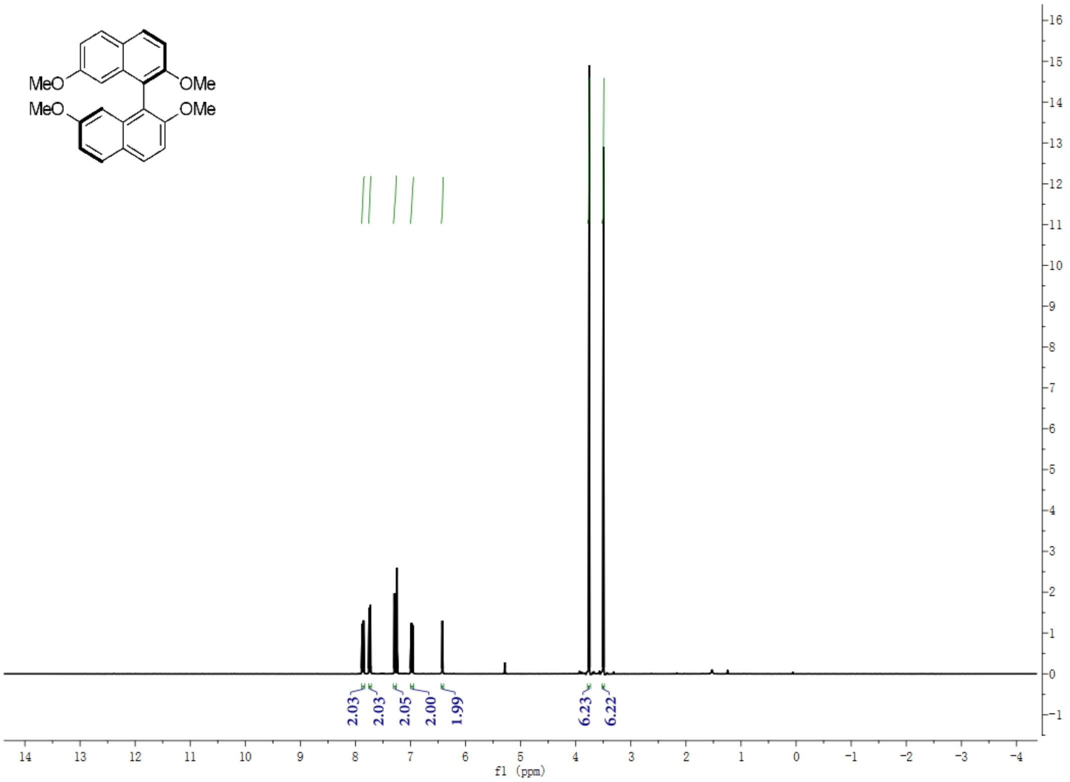

Figure S7. ^{1}H NMR spectrum of **4a**

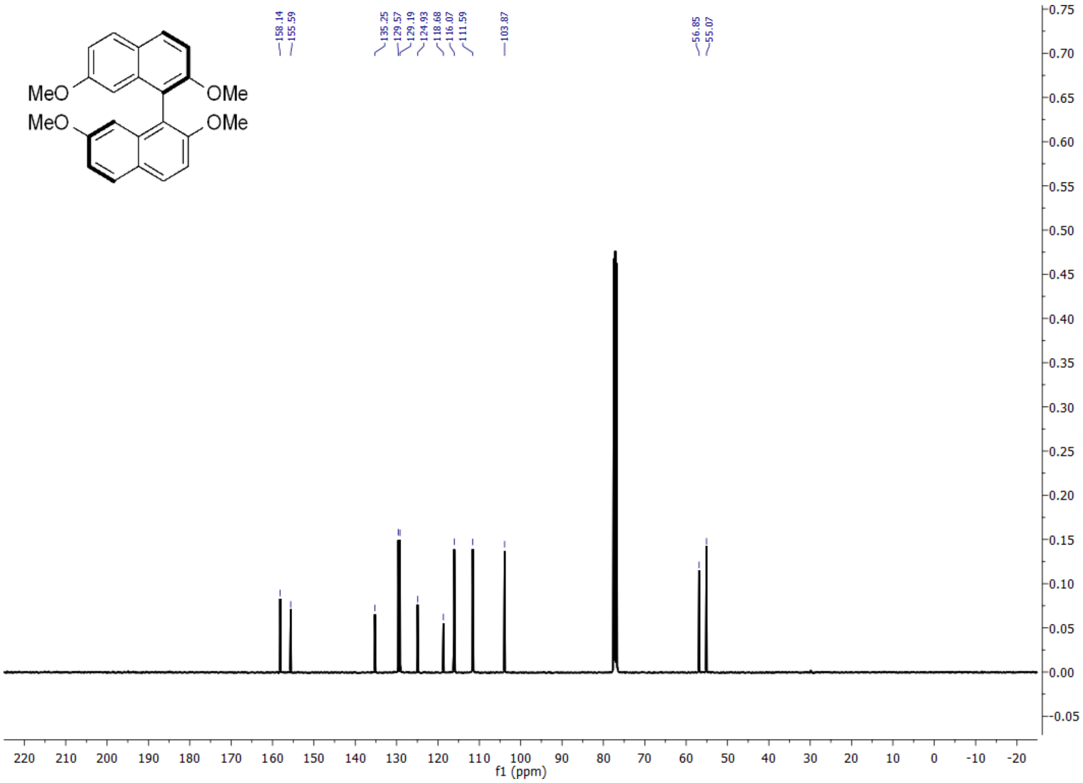

Figure S8. ^{13}C NMR spectrum of **4a**

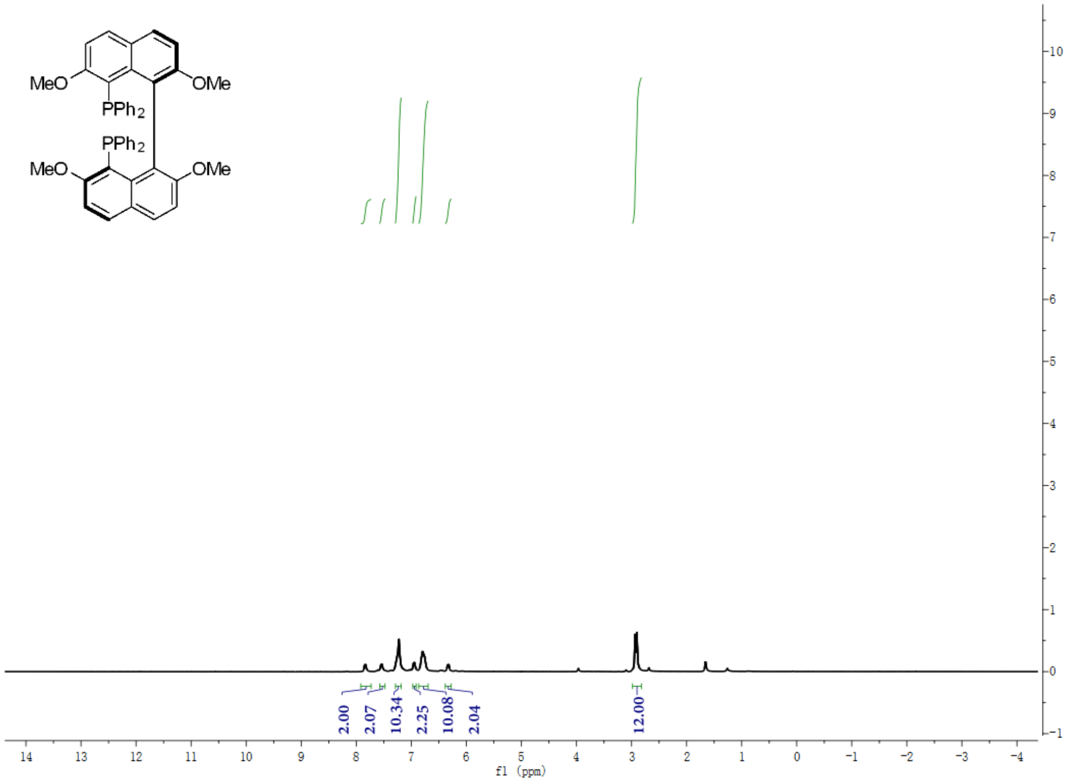

Figure S9. ^{1}H NMR spectrum of **6a**

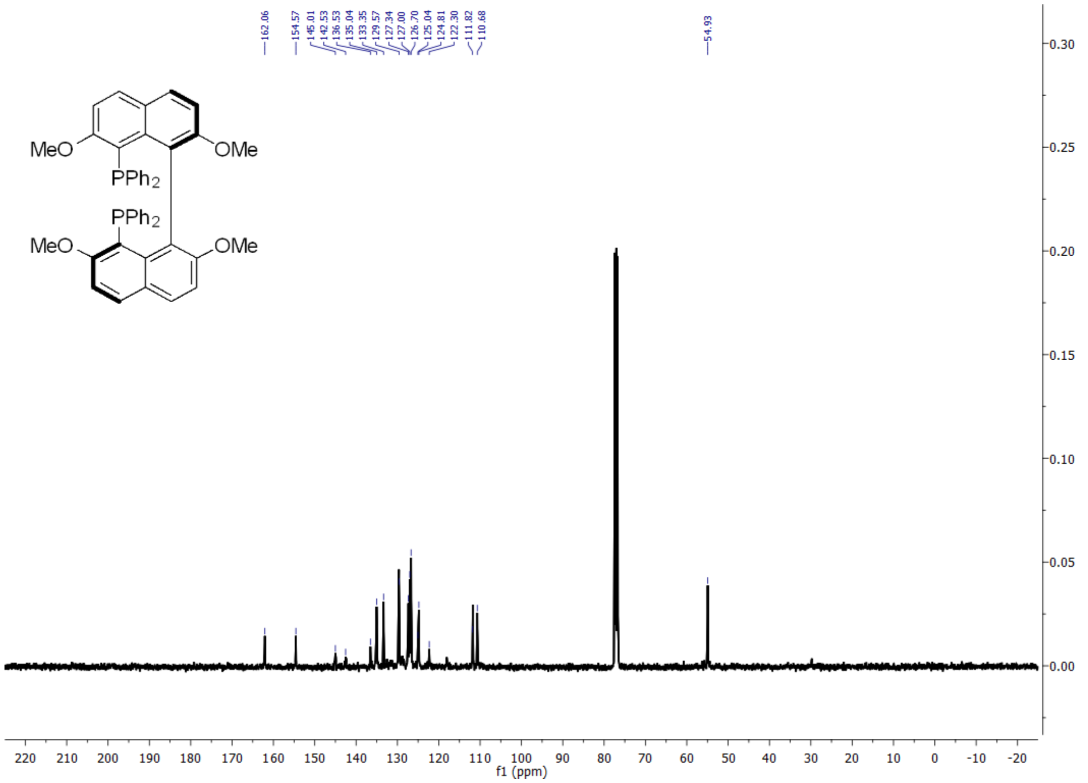

Figure S10. ^{13}C NMR spectrum of **6a**

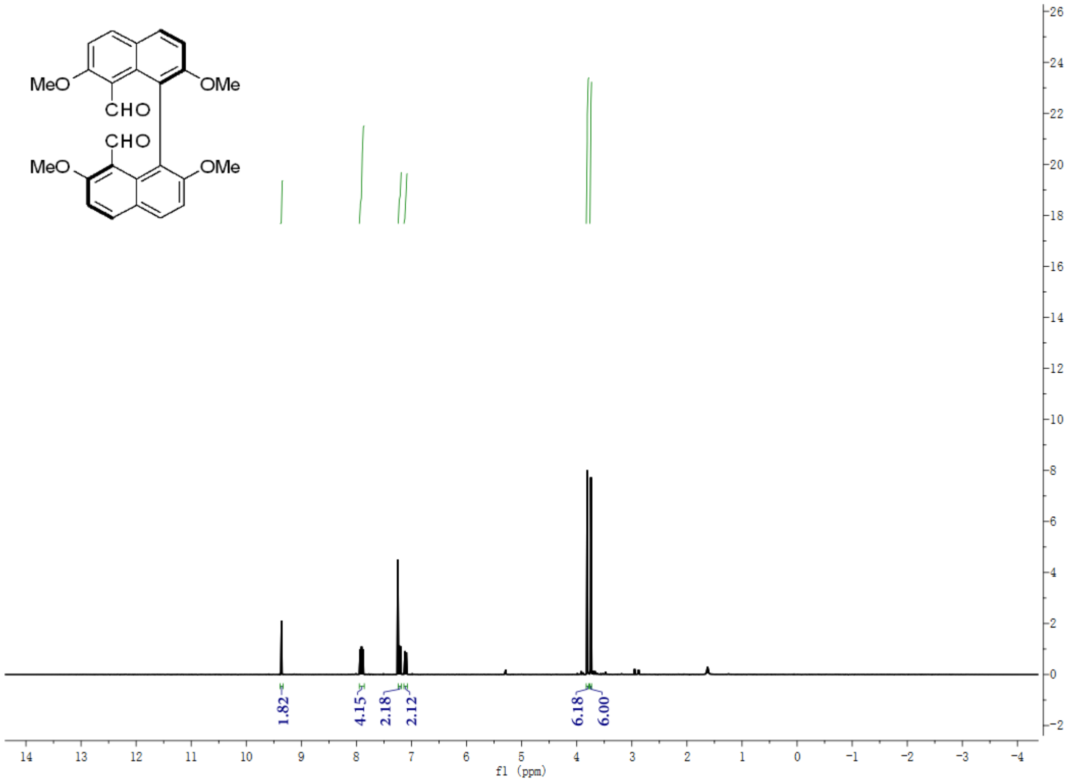

Figure S11. ^{1}H NMR spectrum of **7a**

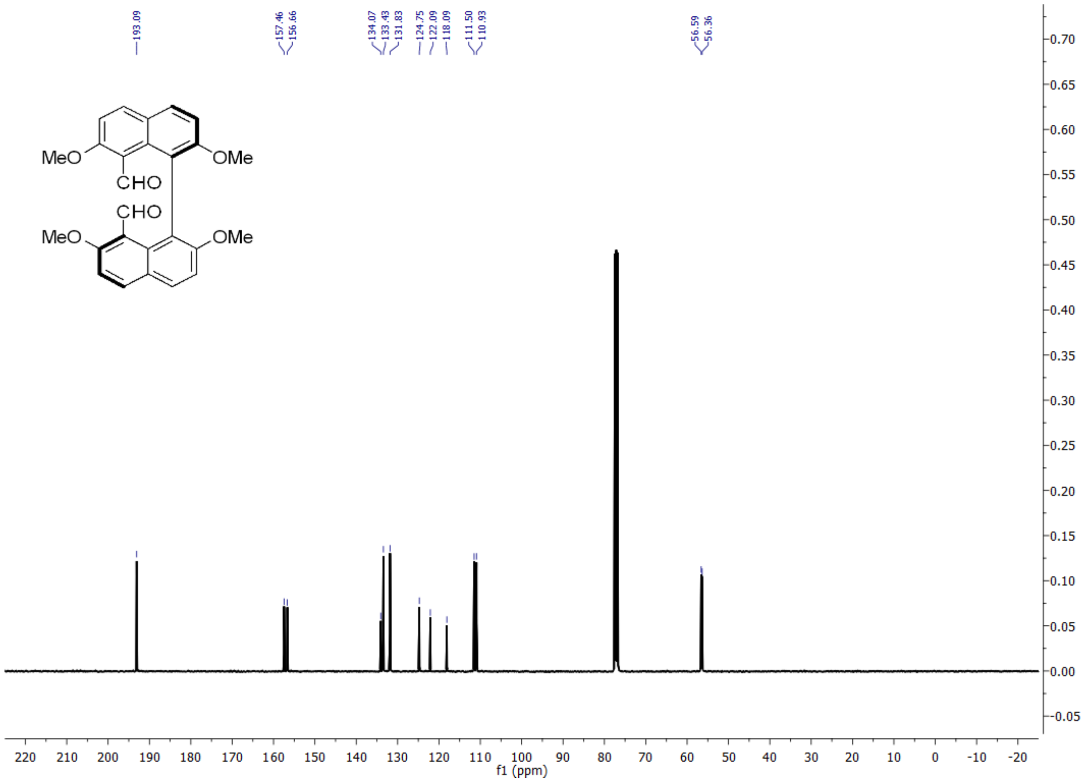

Figure S12. ^{13}C NMR spectrum of **7a**

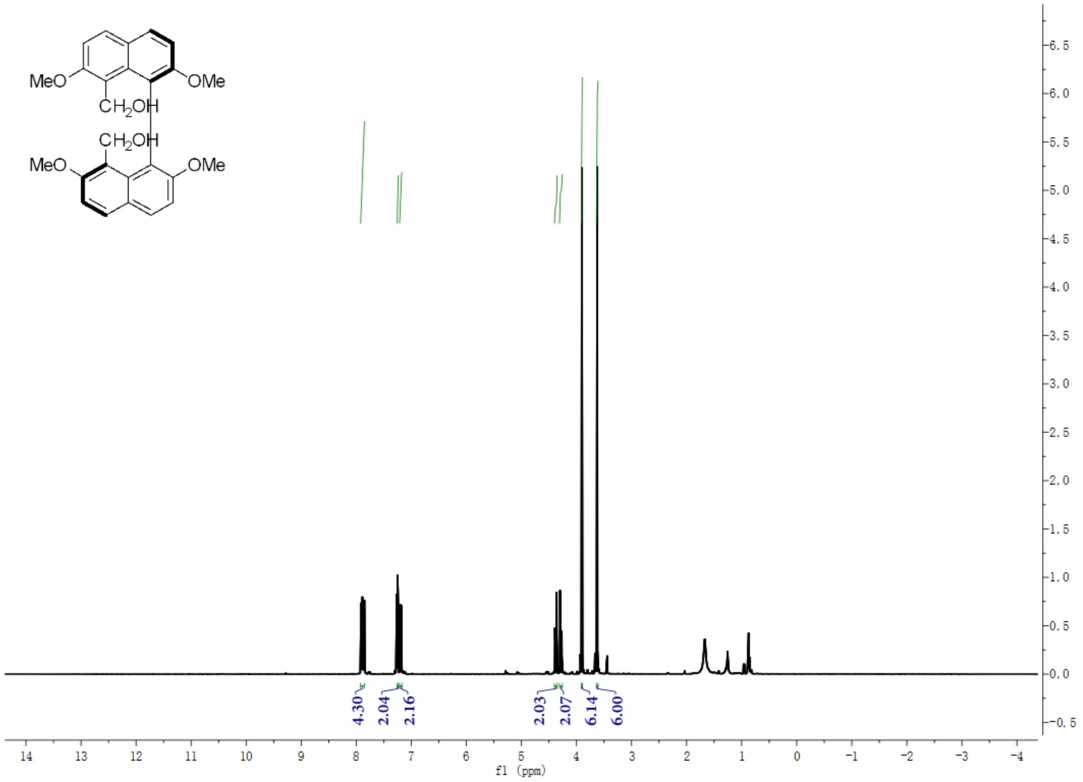

Figure S13. ^{1}H NMR spectrum of **8a**

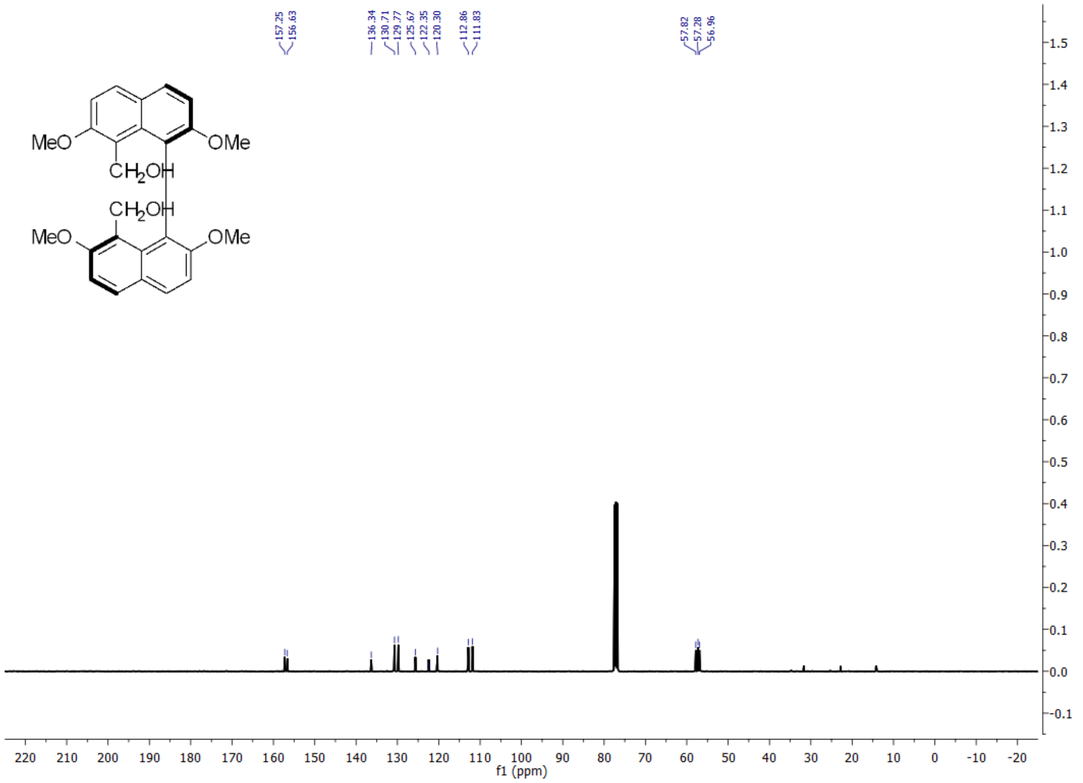

Figure S14. ^{13}C NMR spectrum of **8a**

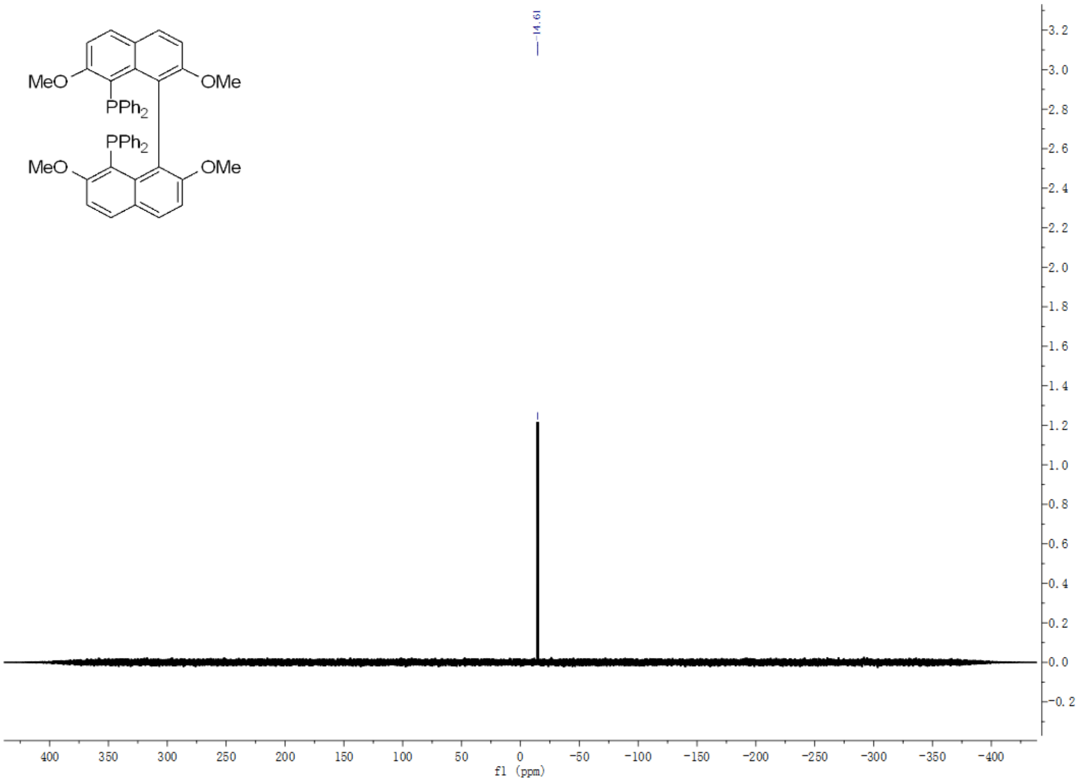

Figure S15. ^{31}P NMR spectrum of **6a**

5. X-ray data

Data were collected on a Rigaku XtaLAB Synergy-*i* Kappa diffractometer equipped with a PhotonJet-*i* X-ray source operated at 50 W (50kV, 1 mA) to generate Cu Kα radiation (λ = 1.54178 Å) and a HyPix-6000HE HPC detector. Crystals were transferred from the vial and placed on a glass slide in type NVH immersion oil by Cargille. A Zeiss Stemi 305 microscope was used to identify a suitable specimen for X-ray diffraction from a representative sample of the material. The crystal and a small amount of the oil were collected on a MiTeGen 50 micron MicroLoop and transferred to the instrument where it was placed under a cold nitrogen stream (Oxford 700 series) maintained at 2400K throughout the duration of the experiment. The sample was optically centered with the aid of a video camera to insure that no translations were observed as the crystal was rotated through all positions.

A unit cell collection was then carried out. After it was determined that the unit cell was not present in the CCDC database a data collection strategy was calculated by *CrysAlis^{Pro}*[1]. The crystal was measured for size, morphology, and color. These values are reported in the accompanying Li21_10_auto_tables file.

Refinement Details

After data collection, the unit cell was re-determined using a subset of the full data collection. Intensity data were corrected for Lorentz, polarization, and background effects using the *CrysAlis^{Pro}*[1]. A numerical absorption correction was applied based on a Gaussian integration over a multifaceted crystal and followed by a semi-empirical correction for adsorption applied using the program *SCALE3 ABSPACK*[2]. The programs *SHELXT*[3] was used for the initial structure solution and *SHELXL*[4] was used for refinement of the structure. Both of these programs were utilized within

the OLEX2 software[5]. Hydrogen atoms bound to carbon atoms were located in the difference Fourier map and were geometrically constrained using the appropriate AFIX commands.

References:

1. CrysAlis^{Pro} (2018) Oxford Diffraction Ltd.
2. SCALE3 ABSPACK (2005) Oxford Diffraction Ltd.
3. Sheldrick, G. M. (2015) *Acta Crystallogr*., C**71**, 3-8.
4. Sheldrick, G. M. (2015) *Acta Crystallogr*., A**71**, 3-8.
5. Dolomanov, O. V.; Bourhis, . L. J.; Gildea, R. J.; Howard, J. A. K.; Puschmann. H. (2009) *J. Appl. Cryst*. **42**, 339-341.

5.1 X-ray Single-crystal Data for Compound **4a**

Datablock: 4a

```
Bond precision:   C-C = 0.0025 A          Wavelength=1.54184

Cell:               a=9.2640(1)       b=10.0749(1)       c=21.4365(2)
                    alpha=90          beta=90            gamma=90
Temperature:        240 K
```

	Calculated	Reported
Volume	2000.75(3)	2000.75(3)
Space group	P 21 21 21	P 21 21 21
Hall group	P 2ac 2ab	P 2ac 2ab
Moiety formula	C24 H22 O4	C24 H22 O4
Sum formula	C24 H22 O4	C24 H22 O4
Mr	374.42	374.41
Dx,g cm-3	1.243	1.243
Z	4	4
Mu (mm-1)	0.677	0.677
F000	792.0	792.0
F000’	794.44	
h,k,lmax	11,12,27	11,12,26
Nref	4221[2415]	4073
Tmin,Tmax	0.877,0.935	0.684,1.000
Tmin’	0.876	

Correction method= # Reported T Limits: Tmin=0.684 Tmax=1.000

AbsCorr = GAUSSIAN

Data completeness= 1.69/0.96 Theta(max)= 76.906

R(reflections)= 0.0304(3874) wR2(reflections)= 0.0812(4073)

S = 1.062 Npar= 258

The following ALERTS were generated. Each ALERT has the format

test-name_ALERT_alert-type_alert-level.

Click on the hyperlinks for more details of the test.

Alert level C

```
PLAT220_ALERT_2_C NonSolvent    Resd 1   C    Ueq(max)/Ueq(min) Range        3.4 Ratio
```

Alert level G

```
PLAT142_ALERT_4_G s.u. on b - Axis Small or Missing .............   0.00010 Ang.
PLAT143_ALERT_4_G s.u. on c - Axis Small or Missing .............   0.00020 Ang.
PLAT912_ALERT_4_G Missing # of FCF Reflections Above STh/L= 0.600      51 Note
PLAT978_ALERT_2_G Number C-C Bonds with Positive Residual Density.      3 Info
```

0 **ALERT level A** = Most likely a serious problem - resolve or explain

0 **ALERT level B** = A potentially serious problem, consider carefully

1 **ALERT level C** = Check. Ensure it is not caused by an omission or oversight

4 **ALERT level G** = General information/check it is not something unexpected

0 ALERT type 1 CIF construction/syntax error, inconsistent or missing data
2 ALERT type 2 Indicator that the structure model may be wrong or deficient
0 ALERT type 3 Indicator that the structure quality may be low
3 ALERT type 4 Improvement, methodology, query or suggestion
0 ALERT type 5 Informative message, check

It is advisable to attempt to resolve as many as possible of the alerts in all categories. Often the minor alerts point to easily fixed oversights, errors and omissions in your CIF or refinement strategy, so attention to these fine details can be worthwhile. In order to resolve some of the more serious problems it may be necessary to carry out additional measurements or structure refinements. However, the purpose of your study may justify the reported deviations and the more serious of these should normally be commented upon in the discussion or experimental section of a paper or in the "special_details" fields of the CIF. checkCIF was carefully designed to identify outliers and unusual parameters, but every test has its limitations and alerts that are not important in a particular case may appear. Conversely, the absence of alerts does not guarantee there are no aspects of the results needing attention. It is up to the individual to critically assess their own results and, if necessary, seek expert advice.

Publication of your CIF in IUCr journals

A basic structural check has been run on your CIF. These basic checks will be run on all CIFs submitted for publication in IUCr journals (*Acta Crystallographica*, *Journal of Applied Crystallography*, *Journal of Synchrotron Radiation*); however, if you intend to submit to *Acta Crystallographica Section C* or *E* or *IUCrData*, you should make sure that full publication checks are run on the final version of your CIF prior to submission.

Publication of your CIF in other journals

Please refer to the *Notes for Authors* of the relevant journal for any special instructions relating to CIF submission.

PLATON version of 28/11/2022; check.def file version of 28/11/2022

Datablock li21_10_auto - ellipsoid plot

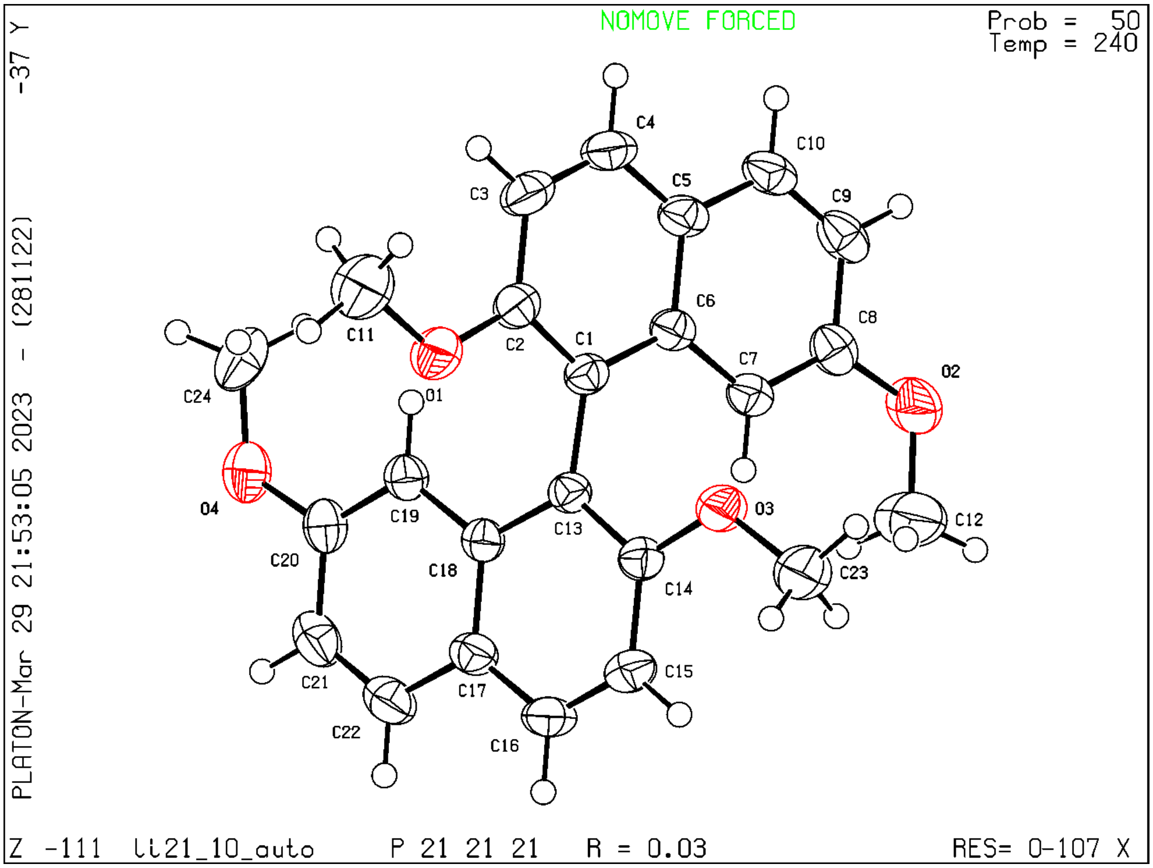

5.2 X-ray Single-crystal Data for Compound ±**6**

Datablock: ±6

Bond precision:	C-C = 0.0023 A		Wavelength=0.71073
Cell:	a=12.0009(2)	b=14.2873(2)	c=14.9328(2)
	alpha=105.545(1)	beta=96.306(1)	gamma=106.849(1)
Temperature:	100 K		

	Calculated	Reported
Volume	2311.50(6)	2311.50(6)
Space group	P -1	P -1
Hall group	-P 1	-P 1

Moiety formula	C48 H40 O4 P2, C7 H8 [+ solvent]	C48 H40 O4 P2, C7 H8
Sum formula	C55 H48 O4 P2 [+ solvent]	C55 H48 O4 P2
Mr	834.87	834.87
Dx,g cm-3	1.199	1.200
Z	2	2
Mu (mm-1)	0.140	0.140
F000	880.0	880.0
F000'	880.76	
h,k,lmax	15,18,18	15,18,18
Nref	9811	9801
Tmin,Tmax	0.968,0.989	0.514,1.000
Tmin'	0.968	

Correction method= # Reported T Limits: Tmin=0.514 Tmax=1.000
AbsCorr = GAUSSIAN

Data completeness= 0.999 Theta(max)= 26.733

R(reflections)= 0.0417(8423) wR2(reflections)= 0.1139(9801)

S = 1.068 Npar= 555

The following ALERTS were generated. Each ALERT has the format

test-name_ALERT_alert-type_alert-level.

Click on the hyperlinks for more details of the test.

Alert level C

DIFMX02_ALERT_1_C The maximum difference density is > 0.1*ZMAX*0.75 The relevant atom site should be identified.

PLAT094_ALERT_2_C Ratio of Maximum / Minimum Residual Density	3.81	Report
PLAT097_ALERT_2_C Large Reported Max. (Positive) Residual Density	1.47	eA-3
PLAT250_ALERT_2_C Large U3/U1 Ratio for Average U(i,j) Tensor	2.1	Note
PLAT910_ALERT_3_C Missing # of FCF Reflection(s) Below Theta(Min).	9	Note

Alert level G

PLAT154_ALERT_1_G The s.u.'s on the Cell Angles are Equal ..(Note)	0.001	Degree
PLAT380_ALERT_4_G Incorrectly? Oriented X(sp2)-Methyl Moiety	C61	Check
PLAT605_ALERT_4_G Largest Solvent Accessible VOID in the Structure	205	A**3
PLAT883_ALERT_1_G No Info/Value for _atom_sites_solution_primary .	Please Do !	
PLAT912_ALERT_4_G Missing # of FCF Reflections Above STh/L= 0.600	1	Note
PLAT978_ALERT_2_G Number C-C Bonds with Positive Residual Density.	13	Info

0 **ALERT level A** = Most likely a serious problem - resolve or explain

0 **ALERT level B** = A potentially serious problem, consider carefully

5 **ALERT level C** = Check. Ensure it is not caused by an omission or oversight

6 **ALERT level G** = General information/check it is not something unexpected

3 ALERT type 1 CIF construction/syntax error, inconsistent or missing data

4 ALERT type 2 Indicator that the structure model may be wrong or deficient

1 ALERT type 3 Indicator that the structure quality may be low

3 ALERT type 4 Improvement, methodology, query or suggestion

0 ALERT type 5 Informative message, check

It is advisable to attempt to resolve as many as possible of the alerts in all categories. Often the minor alerts point to easily fixed oversights, errors and omissions in your CIF or refinement strategy, so attention to these fine details can be worthwhile. In order to resolve some of the more serious problems it may be necessary to carry out additional measurements or structure refinements. However, the purpose of your study may justify the reported deviations and the more serious of these should normally be commented upon in the discussion or experimental section of a paper or in the "special_details" fields of the CIF. checkCIF was carefully designed to identify outliers and unusual parameters, but every test has its limitations and alerts that are not important in a particular case may appear. Conversely, the absence of alerts does not guarantee there are no aspects of the results needing attention. It is up to the individual to critically assess their own results and, if necessary, seek expert advice.

Publication of your CIF in IUCr journals

A basic structural check has been run on your CIF. These basic checks will be run on all CIFs submitted for publication in IUCr journals (*Acta Crystallographica*, *Journal of Applied Crystallography*, *Journal of Synchrotron Radiation*); however, if you intend to submit to *Acta Crystallographica Section C* or *E* or *IUCrData*, you should make sure that full publication checks are run on the final version of your CIF prior to submission.

Publication of your CIF in other journals

Please refer to the *Notes for Authors* of the relevant journal for any special instructions relating to CIF submission.

PLATON version of 28/11/2022; check.def file version of 28/11/2022

Datablock li21_09_auto - ellipsoid plot

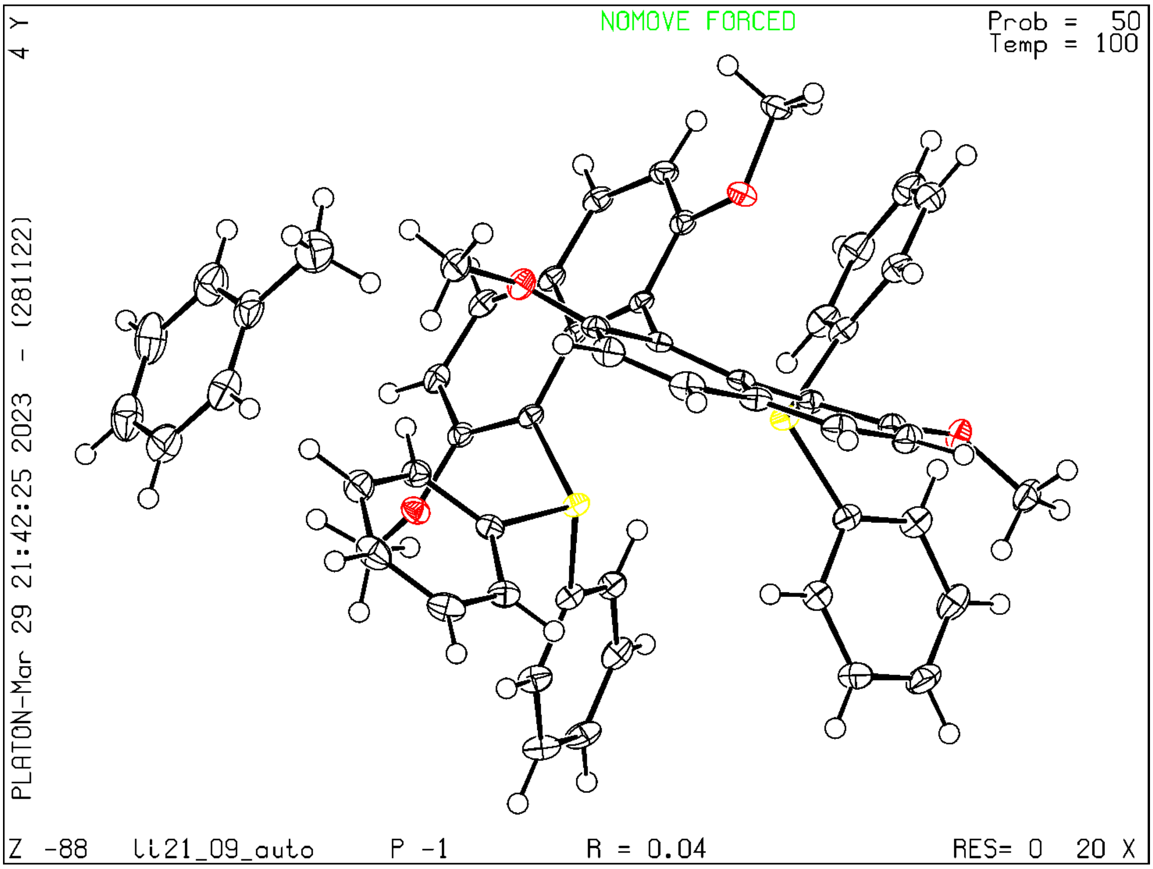

6. HPLC spectrum

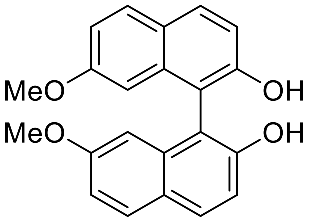

(± 3)

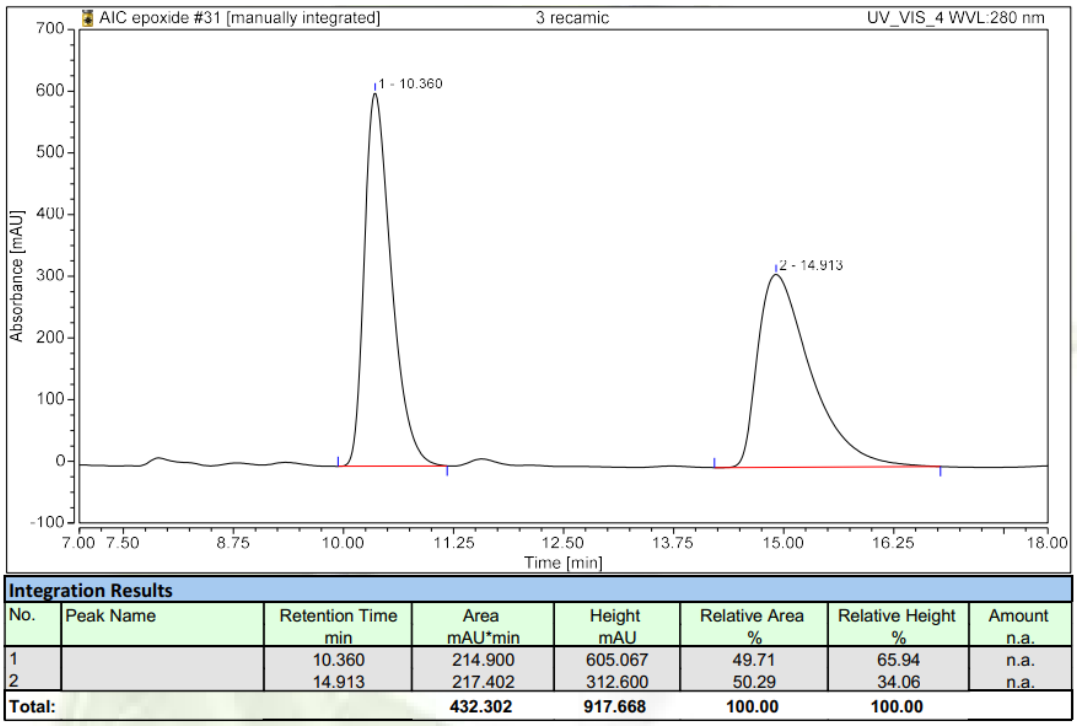

Integration Results							
No.	Peak Name	Retention Time min	Area mAU*min	Height mAU	Relative Area %	Relative Height %	Amount n.a.
1		10.360	214.900	605.067	49.71	65.94	n.a.
2		14.913	217.402	312.600	50.29	34.06	n.a.
**Total:**			**432.302**	**917.668**	**100.00**	**100.00**	

Figure S16. HPLC spectrum of racemic 3.

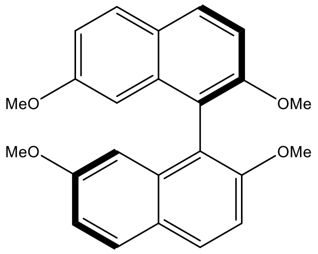

3a, 99% ee

Chromatogram

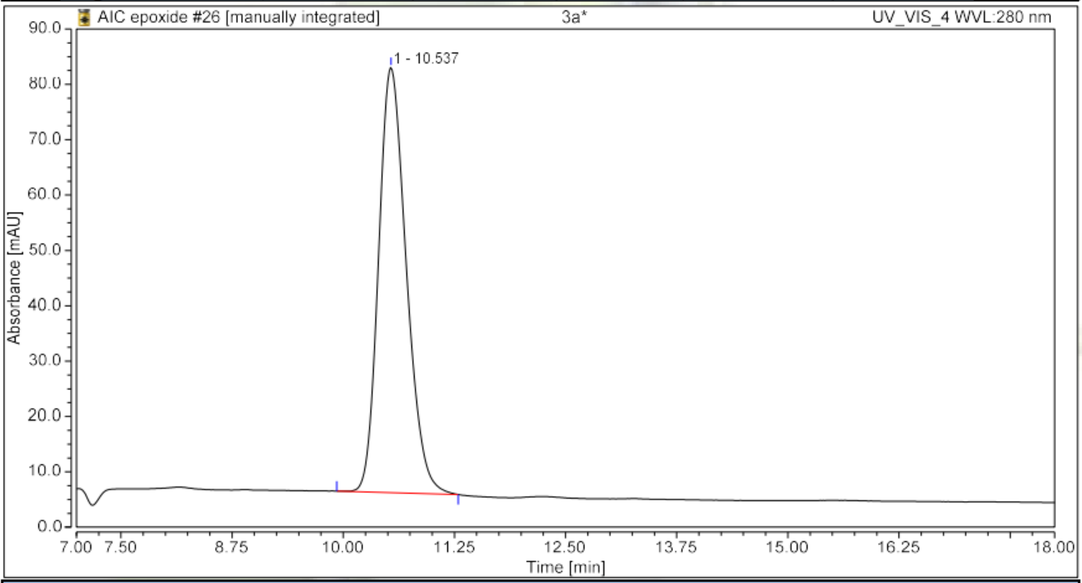

Integration Results

No.	Peak Name	Retention Time min	Area mAU*min	Height mAU	Relative Area %	Relative Height %	Amount n.a.
1		10.537	27.806	76.814	100.00	100.00	n.a.
**Total:**			**27.806**	**76.814**	**100.00**	**100.00**	

Figure S17. HPLC spectrum of 3a.

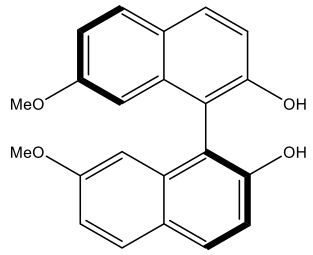

3b, 97% ee

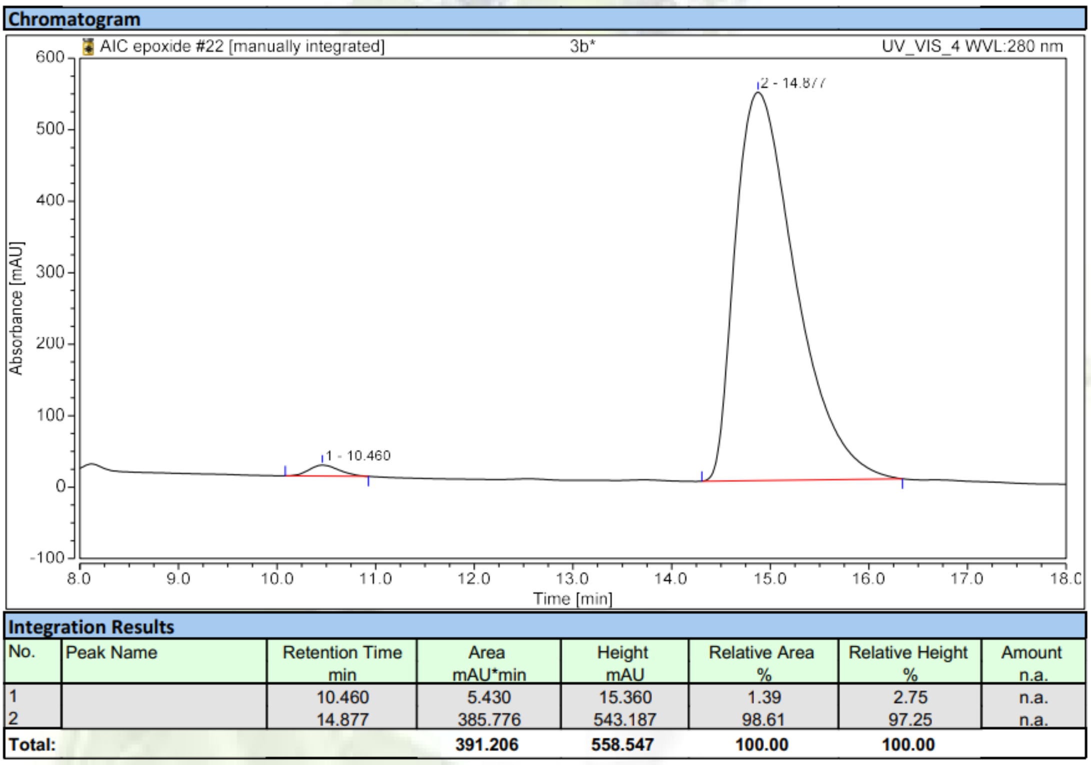

Integration Results

No.	Peak Name	Retention Time min	Area mAU*min	Height mAU	Relative Area %	Relative Height %	Amount n.a.
1		10.460	5.430	15.360	1.39	2.75	n.a.
2		14.877	385.776	543.187	98.61	97.25	n.a.
**Total:**			**391.206**	**558.547**	**100.00**	**100.00**	

Figure S18. HPLC spectrum of 3b.

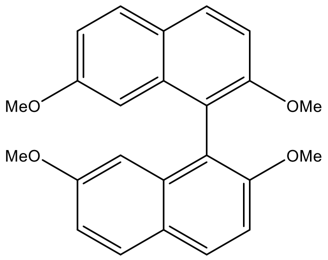

± **4**

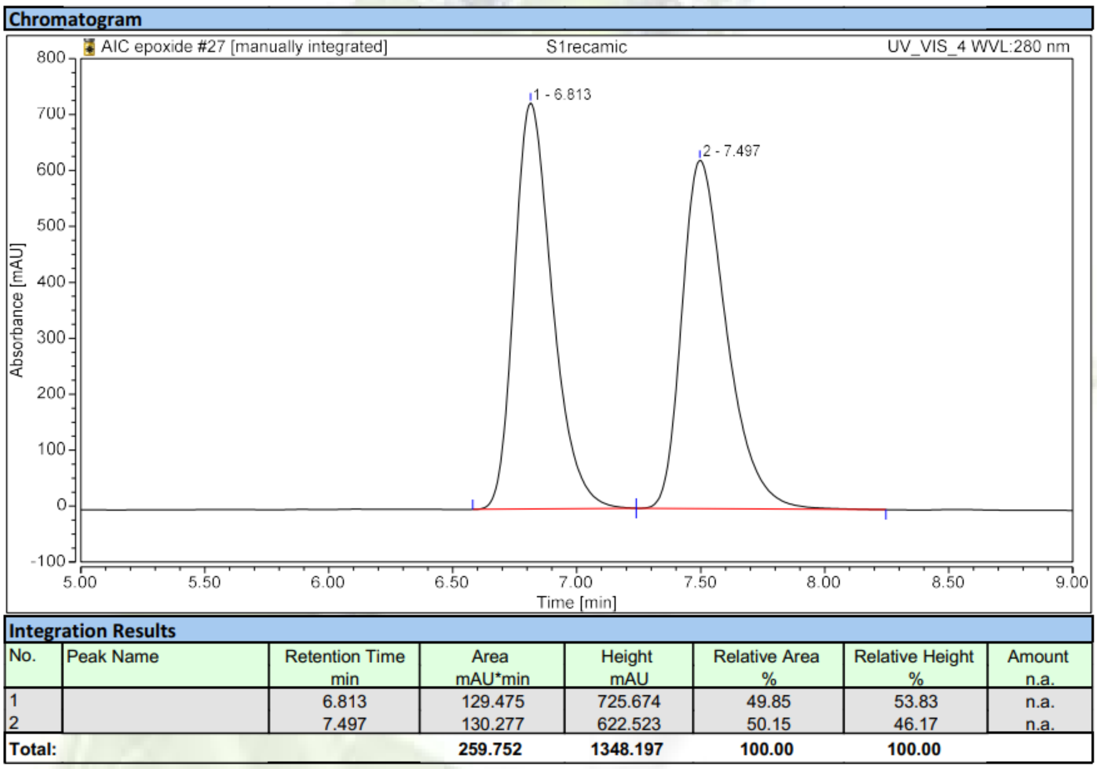

Integration Results

No.	Peak Name	Retention Time min	Area mAU*min	Height mAU	Relative Area %	Relative Height %	Amount n.a.
1		6.813	129.475	725.674	49.85	53.83	n.a.
2		7.497	130.277	622.523	50.15	46.17	n.a.
**Total:**			**259.752**	**1348.197**	**100.00**	**100.00**	

Figure S19. HPLC spectrum of racemic 4.

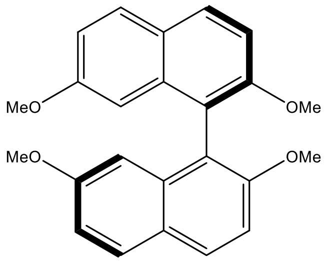

4a, 99% ee

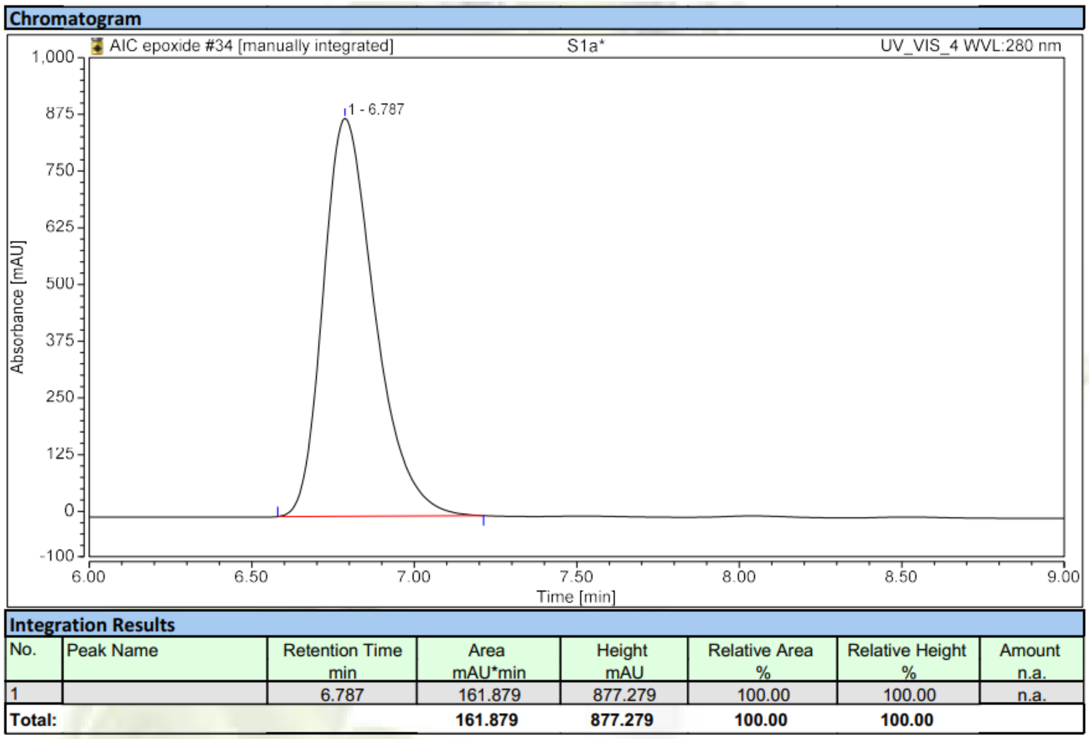

Integration Results

No.	Peak Name	Retention Time min	Area mAU*min	Height mAU	Relative Area %	Relative Height %	Amount n.a.
1		6.787	161.879	877.279	100.00	100.00	n.a.
**Total:**			**161.879**	**877.279**	**100.00**	**100.00**	

Figure S20. HPLC spectrum of 4a.

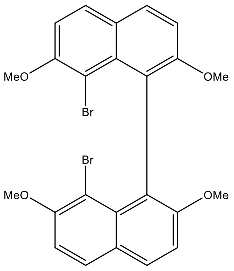

± 5

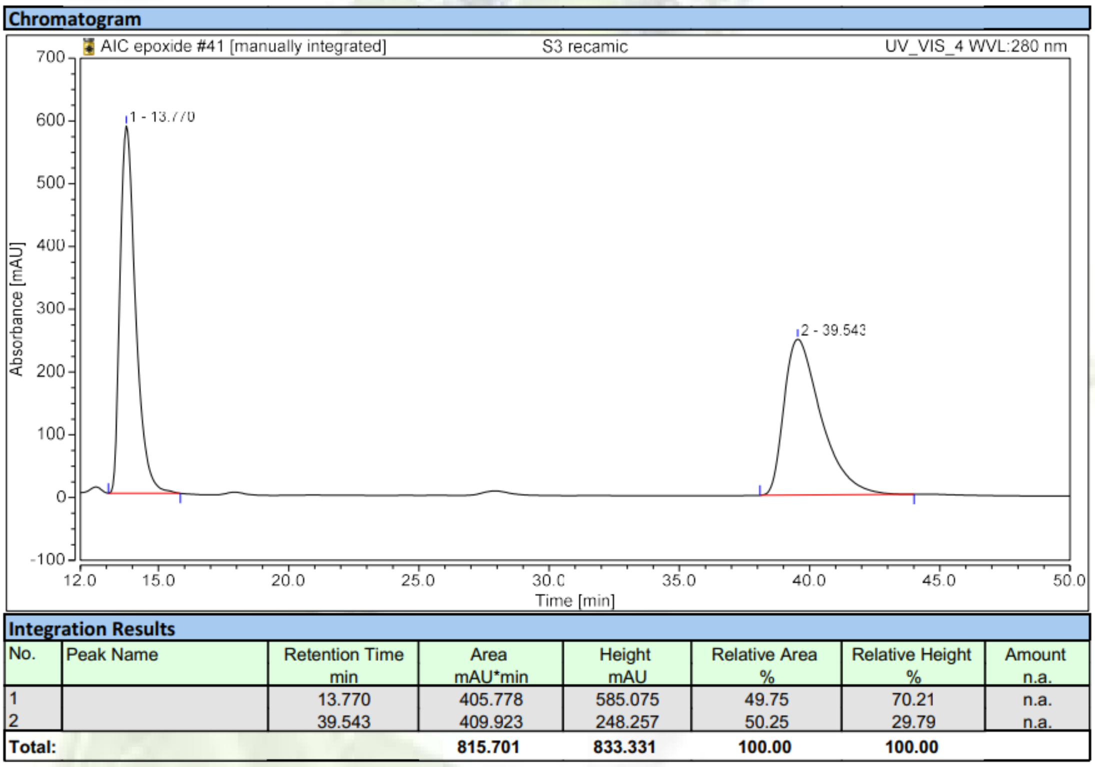

Integration Results

No.	Peak Name	Retention Time min	Area mAU*min	Height mAU	Relative Area %	Relative Height %	Amount n.a.
1		13.770	405.778	585.075	49.75	70.21	n.a.
2		39.543	409.923	248.257	50.25	29.79	n.a.
**Total:**			**815.701**	**833.331**	**100.00**	**100.00**	

Figure S21. HPLC spectrum of racemic 5.

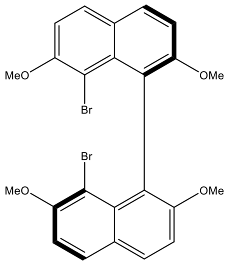

5a, 99% ee

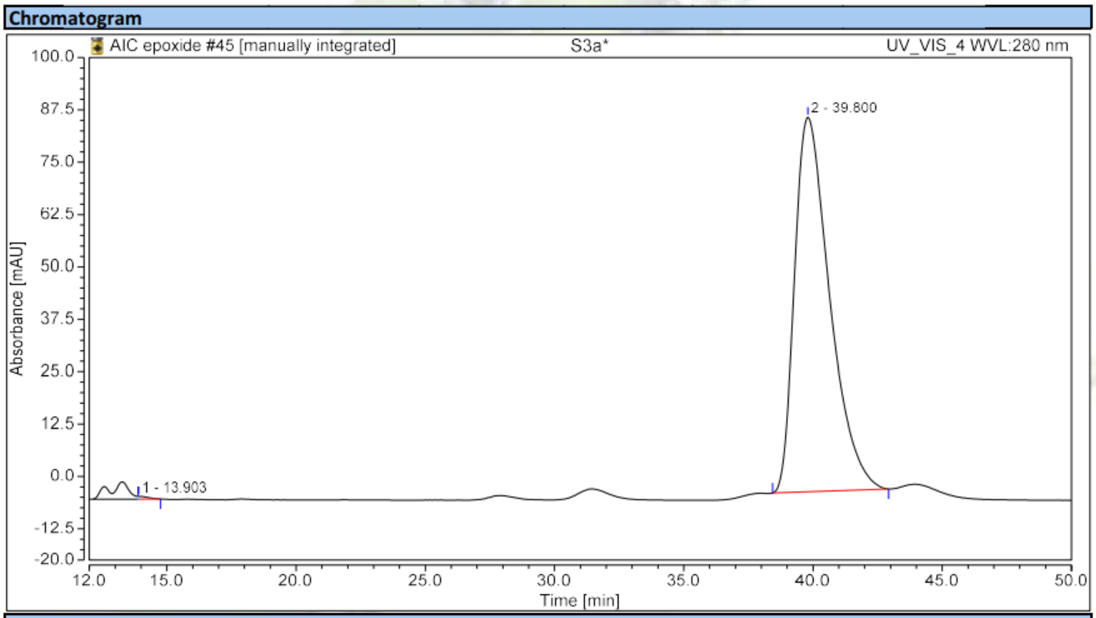

Integration Results

No.	Peak Name	Retention Time min	Area mAU*min	Height mAU	Relative Area %	Relative Height %	Amount n.a.
1		13.903	0.293	0.629	0.21	0.70	n.a.
2		39.800	140.695	89.476	99.79	99.30	n.a.
**Total:**			**140.988**	**90.105**	**100.00**	**100.00**	

Figure S22. HPLC spectrum of 5a.

7. Reference

1. Zhu, Z.; Genaev, A. M.; Salnikov, G. E.; Koltunov, K. Y., Mechanistic investigation of superelectrophilic activation of 1, 1′-bi-2-naphthols in the presence of aluminum halides. *Org. Biomol. Chem*. **2019**, 17, 3971-3977.
2. Che, D.; Andersen, N. G.; Lau, S. Y.; Parvez, M.; Keay, B. A., Synthesis and applications of (R)-and (S)-7, 7′-dimethoxy-2, 2′-bis (diphenylphosphino)-1, 1′-binaphthalene. *Tetrahedron: Asymmetry*. **2000**, 11, 1919-1925.
